# Optomechanical Probes with Tailored Material and Shape Asymmetry Assembled Using DNA Origami

**DOI:** 10.1021/acs.nanolett.5c05354

**Published:** 2026-01-21

**Authors:** David Daniel Ruiz Arce, Markéta Benešová, Václav Protiva, Jaroslav Kočišek, Zdeněk Pilát, Jan Ježek, Lukáš Šilhan, Pavel Zemánek, Alexandr Jonáš, Leo Sala

**Affiliations:** † Department of Dynamics of Molecules and Clusters, J. Heyrovský Institute of Physical Chemistry of the Czech Academy of Sciences, Dolejškova 3, 18200 Prague, Czech Republic; ‡ Department of Microphotonics, 191299 Institute of Scientific Instruments of the Czech Academy of Sciences, Královopolská 147, 61200 Brno, Czech Republic; ¶ Faculty of Chemistry, Brno University of Technology, Purkyňova 118, 612 00 Brno, Czech Republic

**Keywords:** Colloidal Particles, DNA Origami, Optical Trapping, Optomechanics

## Abstract

Optically trapped microscopic probes with precisely defined size, shape, and composition can be used for quantitative environmental sensing and for parametric investigation of fundamental physical phenomena at the classical–quantum boundary. The preparation of uniform ensembles of such probes is challenging, particularly considering probes with controlled shape or material asymmetries. We report a bottom-up strategy for fabricating the optomechanical probes using DNA nanotechnology. Specifically, we synthesize Janus-type colloidal heterodimers comprising two microspheres of different materials and sizes interconnected by 24HB DNA origami nanostructures. The interconnecting DNA origami scaffolds both facilitate the heterodimer assembly and enable their functionalization with other optical components. The utility of the fully assembled probes is then demonstrated by their 2D and 3D manipulation with optical tweezers. The versatility of the presented approach opens up the way toward fabricating novel custom-tailored probes for optomechanical experiments.

## Graphical abstract

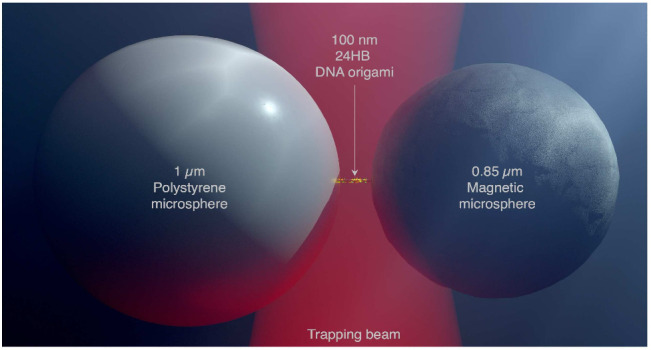

Micro- and nanoobjects with a precisely controlled size, shape, and optical characteristics have attracted growing interest for diverse applications, from miniature translational and rotational motors driven by light^1,2^ through plasmonic and photonic structures with tailored spectral responses^3,4^ to custom probes for optomechanical experiments.^5,6^ To provide the desired functionality, such systems rely on a well-defined spatial arrangement of their components represented by dielectric and metal micro- and nanoparticles, quantum dots, nanodiamonds, or fluorescent molecules.^7,8^ Traditionally, they have been produced using top-down techniques,^9^ facing limitations related to the speed of fabrication, requirement of complex and expensive equipment, and difficulty in producing hybrid micro- and nanostructures with different elements arranged in close proximity with a well-defined stoichiometry.

Bottom-up self-assembly of complex microstructures from elementary parts offers an elegant way for circumventing the above shortcomings.^3^ Among the various strategies, DNA origami nanostructures (DON)^10,11^ provide unique benefits to bottom-up fabrication, such as the high control over the shape of the self-assembled structures and the addressability of their individual molecular components. Fabrication of micron-sized objects based on DON is an active field of research.^12,13^ Recent breakthroughs in the field are represented by the crystalline DON assemblies,^14,15^ having the potential to significantly advance the field of optical metamaterials that possess a photonic bandgap. However, the “all-DNA” path to microscale objects faces several challenges, such as the material cost, design complexity, duration of the self-assembly process, and DNA stability. In various applications, including optomechanics and light-driven actuation, these issues could be avoided by combining DON with larger particles made of other materials to form the target objects.

Hybrids of DON with individual microparticles were prepared for DON purification,^16,17^ programmable DON assembly,^18^ manipulation of DON attached to a substrate,^19,20^ or studies of magnetically driven microscale propulsion.^21^ The attempts to link two or more microscale objects using DON, however, have only been reported in connection with two specific applications to date. In the single-molecule force spectroscopy of DNA using optical tweezers, DON linked to two optically trapped microparticles that served as force transducers were first mechanically characterized^22^ and then employed to probe the DNA stacking interactions.^23^ In the field of magnetically actuated microswimmers,^24^ emerging in biomedical applications, heterodimers of magnetic and polystyrene microparticles were prepared by interconnecting the two constituent particles immobilized on a template by a DON interlink.^25^ A similar approach was also used in ref 26 to create more complex architectures of environmentally responsive microswimmers.

In all these studies, only two methods have been used to prepare hybrid microstructures: (i) one by one assembly by controlled manipulation of the constituent microparticles (e.g. refs 22 and 23 ), which provides high control over the composition of the synthesis product, but its yield is extremely low and (ii) template-assisted assembly,^25,26^ which has significantly higher yield, but requires the “top-down” preparation of the assembly template reducing design flexibility and adding complexity to the fabrication process. In this paper, we present a batch synthesis of heterodimers from different types of colloidal microparticles using DON interlinks. The selective coupling of the constituent microparticles is achieved by the interlink design, eliminating the need for microparticle manipulation or immobilization on a template. Consequently, the method is simpler and more flexible than the previously reported methods, well-suited for the preparation of uniform ensembles of optomechanical probes. An additional advantage of using the DON interlinks is the ability to position functional molecules, such as fluorescent dyes, at predefined nanometer-scale locations, enabling multiplexed architectures difficult to achieve with more conventional DNA-mediated assembly techniques.^27,28^ The choice of the constituent microparticles and fluorescent labels for the present work is driven by the specific application targeting the use of the synthesized heterodimers as custom optomechanical probes whose shape and material asymmetry can be exploited for quantitative environmental sensing using optical trapping.^5,6^ We optimize the individual steps of the probe synthesis, characterize the structural integrity and fluorescence properties of the resulting product, and evaluate the feasibility and limitations of its deployment in optical manipulations.

## Heterodimer Design

We introduce a method for fabricating optomechanical probes with complex shapes and material composition by assembling microspheres using DON interlinks. To demonstrate the versatility of such an approach, we prepared heterodimers of Janus type^29^ by binding 1 μm polystyrene microspheres and 0.85 μm magnetic Magnefy microspheres (Figure 1a), which can be employed in micromanipulation experiments. In particular, the polystyrene component provides an excellent optical trapping efficiency in aqueous environments, while the magnetic component could enable another mode of manipulation for further applications. The asymmetric shape and material composition of the heterodimer probes can then be used for their dynamic orienting in optical fields.

**Figure 1:**
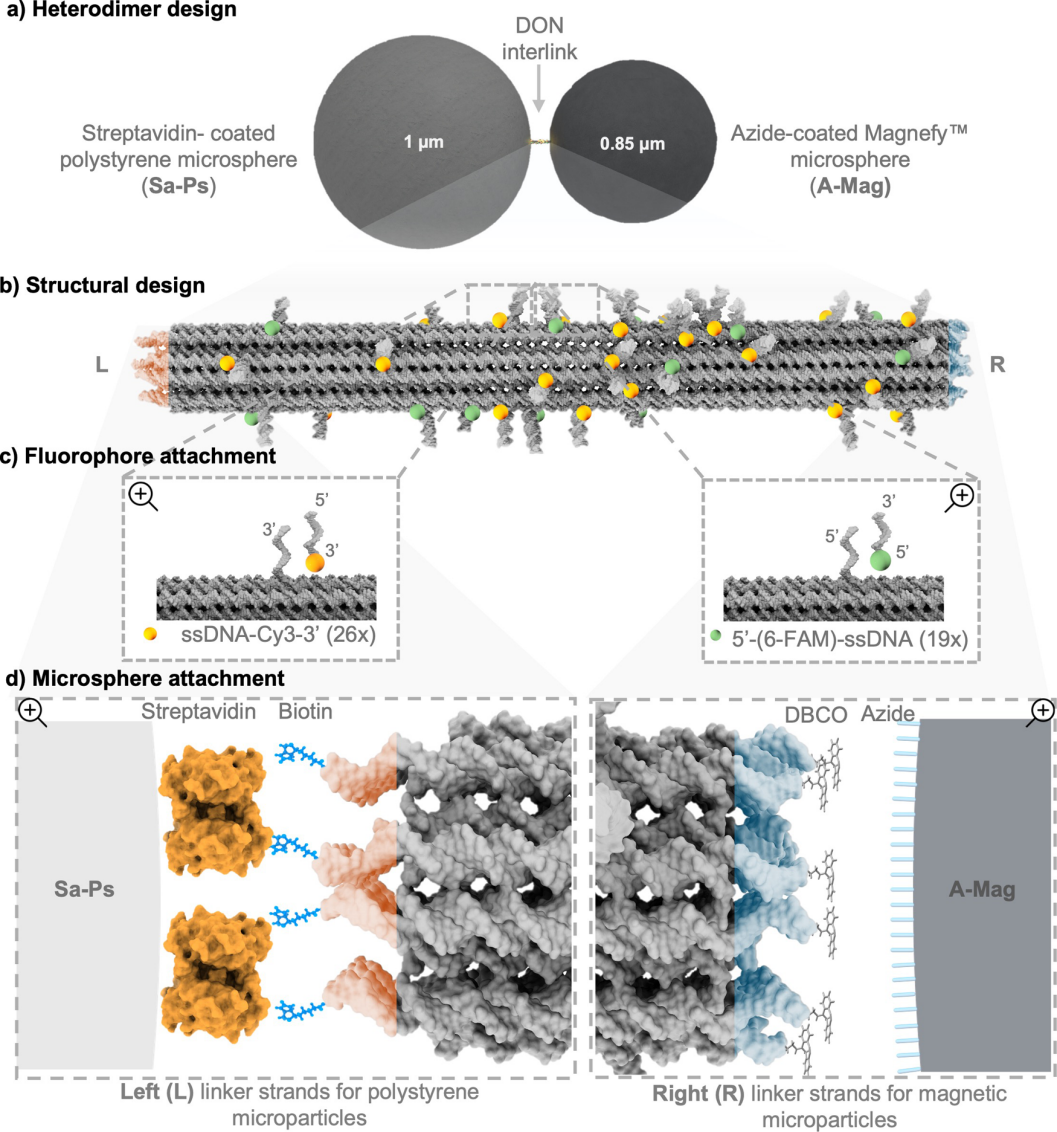
Heterodimer design. (a) Sketch of the target heterodimer structure consisting of streptavidin-coated polystyrene (Sa–Ps) and azide-coated Magnefy (A–Mag) microspheres interconnected by the DON interlink. (b) A 3D representation of the 24HB DON interlink, showing the binding sites for attaching the fluorescent dyes and the linker strands to the microspheres at the left (L) and right (R) ends. (c) An illustration of the fluorophore attachment strategy, highlighting the 6-FAM (green) and Cy3 (orange) dyes. (d) A 3D representation of the microsphere linker sites for the streptavidin–biotin (L) and azide–DBCO (R) conjugation. The images are not drawn to scale. See Section 2 of the Supporting Information (SI) for additional details.

To assemble our optomechanical probes, we adapted a 100 nm long 24-helix bundle (24HB) DNA origami having a cross-sectional diameter of 16 nm based on the original design of Kuzyk et al.^30^ (see Figure 1b). This platform provides high structural stability suitable for the present application. To allow site-specific conjugation, we designed single-stranded DNA (ssDNA) overhangs in the 24HB at its left (L) and right (R) end regions by extending 14 staples at either the 5′ or 3′ ends with polythymine (poly-T) nucleotide segments. The outer terminus for each modified staple strand was functionalized with biotin at the L-end and with dibenzocyclooctyne (DBCO) at the R-end, enabling site-specific conjugation of the two microspheres (see Figure 1d). The difference in the size of the constituent microspheres allowed us to distinguish the heterodimers assembled via the specific 24HB interlinks from the homodimers that may also be formed via spontaneous microsphere coagulation. Further verification of the specificity of microsphere coupling was then enabled by the fluorescent dye functionalization of the interlinking 24HB.

To demonstrate the functionalization capabilities of our platform, the 24HB interlinks were modified with 6-carboxyfluorescein (6-FAM) and Cy3 fluorescent dyes. We employed an “external labeling” strategy, where the fluorophores were fixed at specific binding sites close to the 24HB surface. To implement this, 26 staples were extended at the 3′ end to attach to ssDNA-Cy3–3′ oligonucleotides and 19 staples were extended at the 5′ end to attach to 5′-(6-FAM)-ssDNA oligonucleotides bearing the desired sequence complementary to the associated binding sites (see Figure 1b and Experimental section of the SI for details).

## Synthesis and Validation

The detailed synthesis protocol is described in SI Experimental Section 1.2. Briefly, 24HB were first self-assembled, incorporating all extended staples and ssDNA–dye conjugates during the annealing process. To minimize aggregation caused by multiple extended ssDNA binding sites, we tested three concentrations of MgCl_2_ in the folding buffer (FOB),^31^ finding 20 mM MgCl_2_ to be the optimum with respect to the uniformity in the 24HB morphology and reduced aggregation of the final product, as probed by atomic force microscopy (AFM) (see Figures S5–S8). Unless explicitly stated otherwise, all subsequent experiments were conducted using this concentration of MgCl_2_ in the FOB. The successful folding and functionalization of 24HB with the fluorescent dyes were further confirmed by transmission electron microscopy (TEM) and fluorescence microscopy (FM), respectively (see Figures S18 and S9).

In the next step, the 24HB scaffolds were hybridized to microspheres with two different surface chemistries: streptavidin-coated polystyrene microspheres (Sa–Ps) [diameter (1.00 ± 0.05) μm] and azide-coated Magnefy microspheres (A–Mag) [diameter (0.85 ± 0.10) μm] (commercially available from Bangs Laboratories, Cat. No. CP01004 and CBMFY01a, respectively). First, the efficiency of binding of 24HB to each microsphere type was tested individually upon incubation with a 100-fold excess of 24HB (see Figures S12 and S13). The fluorescence emission from the microspheres without and with the attached fluorescently labeled 24HB was observed at the excitation wavelengths of 365 nm (UV channel), 460 nm (6-FAM channel), and 550 nm (Cy3 channel). Spatially confined emission from discrete locations at the microsphere surface, excited at 460 and 550 nm, confirmed the presence of 6-FAM and Cy3, respectively. These specific fluorescence signals from the attached dyes were dominated by Cy3, which is consistent with its higher brightness and photostability at a slightly basic pH of the working buffer. Nonspecific bulk emission excited at 365 nm then revealed that the two types of microspheres could be distinguished by their autofluorescence. In particular, the streptavidin-coated polystyrene microspheres displayed strong autofluorescence signal in the UV excitation channel, whereas no emission was detected from the azide-coated magnetic microspheres in this channel (see Figures S10 and S11).

Figure 2 shows FM and TEM images of the synthesized heterodimers assembled using the 1:1:100 mixing ratio of polystyrene microspheres to magnetic microspheres to 24HB. FM reveals spots of intense Cy3 emission localized at the perimeter of the microspheres, including the contact areas between the individual particles in multimers, which indicates the presence of 24HB nanostructures covering and bridging the microspheres. For the dimers identified in the bright field image (leftmost panel of Figure 2a), correct heterodimer formation could be verified by observing the autofluorescence of the constituent microspheres in the UV channel, where only the polystyrene microspheres were visible. The presence of 24HB at the heterodimers’ connection points was further verified using TEM (see Figure 2b). One can see that the 24HB structures protrude away from the surface only at the point of contact between the constituent microspheres where the 24HBs are bound to both microspheres. At all other locations across the surface, 24HBs lie parallel to the surface, suggesting that upon drying the particles for TEM analysis, this configuration is energetically favored in the absence of a binding partner (see Figures S19 and S20). TEM imaging, therefore, provides unambiguous visual confirmation of the successful specific binding of both constituent microspheres forming the heterodimers to 24HB.

**Figure 2:**
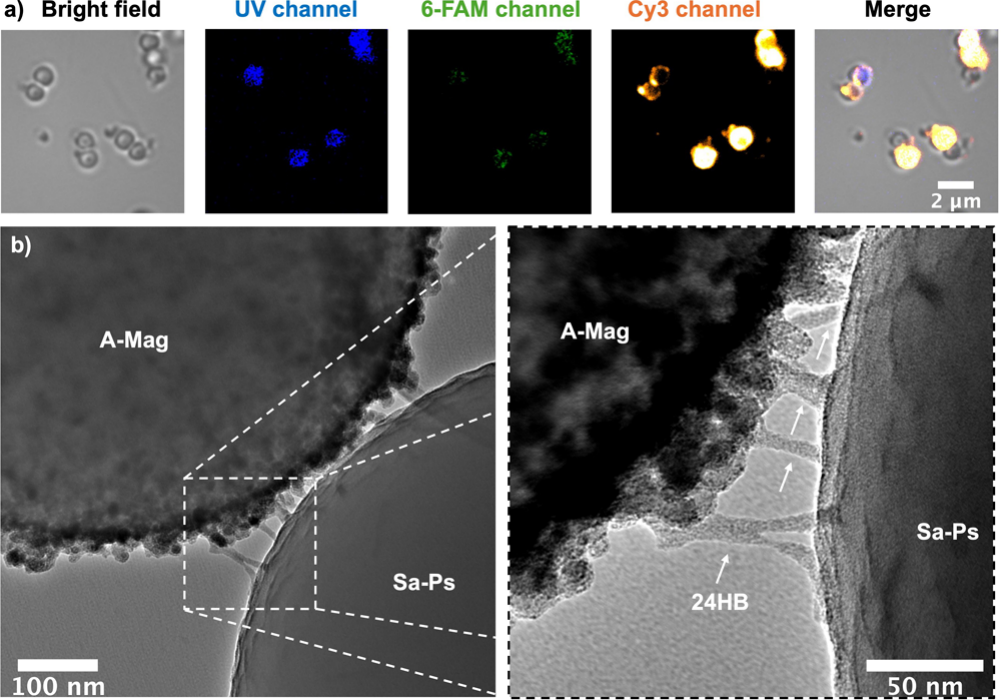
(a) Optical microscopy images of the 1:1:100 heterodimer structures labeled with Cy3 and 6-FAM. “UV channel”, “6-FAM channel”, and “Cy3 channel” correspond to 365, 460, and 550 nm fluorescence excitation wavelengths, and 420–460 nm, 495–540 nm, and 570–625 nm emission detection windows, respectively. Autofluorescence of the polystyrene microspheres can be seen in the UV channel. (b) TEM image of a representative formed heterodimer including a magnified view of the area between the constituent microspheres showing the 24HB bridging the microspheres. A–Mag: Magnefy microsphere, Sa–Ps: streptavidin-coated polystyrene microsphere.

Figure 3 summarizes the results of the analysis of the effect of the microsphere:DNA ratio on the yield of the heterodimer synthesis. The proportion of the correctly assembled heterodimers increases with the excess 24HB: ∼4.4% at 1:1:1, ∼6.5% at 1:1:10, and ∼15% at 1:1:100 microsphere-to-24HB mixing ratio. The 1:1:100 microsphere-to-24HB ratio provides a large excess of 24HB linkers relative to the available microsphere binding sites, ensuring that each microsphere has a high probability of encountering multiple DNA origami structures during the incubation time and, subsequently, forming heterodimers. At a lower excess of 24HB (1:1:10 and 1:1:1), the system becomes linker-limited. This results in (i) an increased overall fraction of unbound microspheres, (ii) a lower probability of DON-induced dimerization (heterodimers) in comparison to nonspecific dimerization (homodimers), and (iii) a reduced likelihood that both ends of a given 24HB will find the correct complementary microsphere. It is worth mentioning that at the 1:1:1 microsphere-to-24HB ratio, the correctly assembled heterodimers show fluorescence emission located predominantly at the contact point between the two constituent microspheres (see Figure 3 and Figure S17), which can be advantageous in some applications, despite the lower synthesis yields. Overall, Figure 3 indicates that the improved yield of heterodimer synthesis at the 1:1:100 microsphere-to-24HB ratio is not only a consequence of more frequent interactions of the DNA with the microspheres but also reflects a shift in the balance between productive and nonproductive interactions in favor of the heterodimer formation (see Figure S15). Therefore, the 24HB nanostructures actively mediate the assembly process. This makes the system promising for further optimization toward higher efficiency of the heterodimer formation. For example, slow annealing protocols^27,32^ can change the binding extension accessibility and the diffusion of the particles in the solution, both affecting the heterodimer assembly. Furthermore, the linker presaturation, in which each microsphere population is functionalized with excess DNA origami before mixing, can lead to suppression of uncontrolled aggregate formation. Such an approach was successfully used for gold nanoparticle assemblies^33,34^ reporting yields of formation of gold nanoparticle dimers as high as 26%.^35^

**Figure 3:**
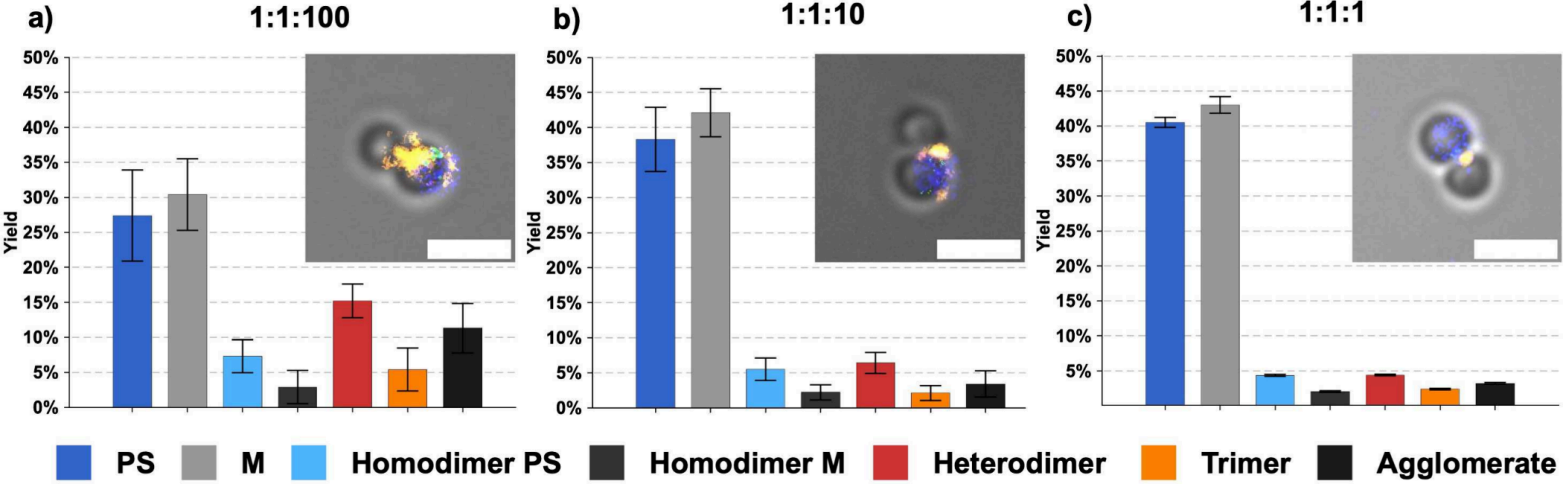
Quantitative yield analysis of the heterodimer formation at different microsphere-to-24HB ratios, based on 10 images of each studied sample (field of view approximately 88.9 μm × 50.0 μm). A total of 2041 objects (1:1:1), 1866 objects (1:1:10), and 1093 objects (1:1:100) were analyzed. Error bars represent standard deviation. Image insets show representative merged fluorescence images of heterodimers formed at the corresponding ratios. Scale bars: 2 μm (see also Figures S15–S17).

Finally, we tested the response of heterodimers to a magnetic field using a permanent magnet. Details can be found in section 1.9 of the SI. The supporting video “Magnetic manipulation” illustrates the response of a heterodimer suspended in the working buffer, whose polystyrene microsphere adheres to the surface, to the oscillatory motion of the magnet across the sample. The magnetic microsphere follows the motion of the magnet, pivoting back and forth around the adherent polystyrene microsphere. Magnetic actuation is then even more pronounced in a large asymmetric cluster containing multiple magnetic microspheres visible next to the heterodimer. This proof-of-principle experiment confirms that the synthesized probes can be manipulated with magnetic forces.

## Optical Manipulation of Synthesized Heterodimers

Optical manipulation of the synthesized colloidal heterodimers was carried out using a linearly polarized single-beam optical trap (optical tweezers) that was generated by tight focusing of an infrared laser beam (wavelength 1064 nm, total power at the sample plane 25–35 mW) with a high numerical aperture microscope objective (see section 1.8 of the SI for details).

In general, microscopic objects with a shape or material asymmetry experience optically induced torques upon illumination with the trapping light. These torques then lead either to stable spatial reorientation of the object with respect to the beam propagation and polarization directions or to sustained rotation of the object.^6^ Heterodimers formed from a polystyrene microsphere (refractive index *n*_PS_ = 1.58 + 1.2 × 10^–3^i at 1064 nm,^36^ diameter (1.00 ± 0.05) μm) and a magnetic microsphere (refractive index *n*_Mag_ = 1.63 + 2.4 × 10^–3^i at 1064 nm,^37^ diameter (0.85 ± 0.10) μm) display both material and shape asymmetry. In a linearly polarized trapping beam focused to a diffraction-limited spot with the diameter comparable to the diameters of the constituent microspheres, such heterodimers are trapped with their longest dimension oriented along the beam propagation direction, as this configuration minimizes the overall energy of the light–matter interaction.^6^

Figure 4a illustrates the full 3D optical manipulation of a heterodimer by the focused laser beam propagating along the *z*-axis, with the focal plane placed in the bulk liquid, sufficiently far away from the walls of the sample chamber. As expected, this arrangement resulted in the all-optical confinement of the heterodimer with its long axis aligned with the beam propagation axis [panel (i)]. Since the sample was observed along the *z*-axis, only one of the two constituent microspheres was visible, obscuring the other one. Stability of the confinement was then demonstrated by translating the whole sample chamber past the stationary optical trap using the microscope stage. During this process, the trapped heterodimer remained fixed in the field of view, despite the hydrodynamic force exerted on it by the moving liquid. On the contrary, a reference object freely diffusing in the liquid was observed to move along the *y*-axis, together with the sample chamber [compare panels (i) and (ii)]. Finally, when the trapping beam was turned off, the confinement was lost and the heterodimer was free to diffuse in the liquid, randomly changing its orientation, which made both constituent microspheres intermittently observable [see panels (iii), (iv), and the supporting video “3D Dimer Trapping”].

**Figure 4:**
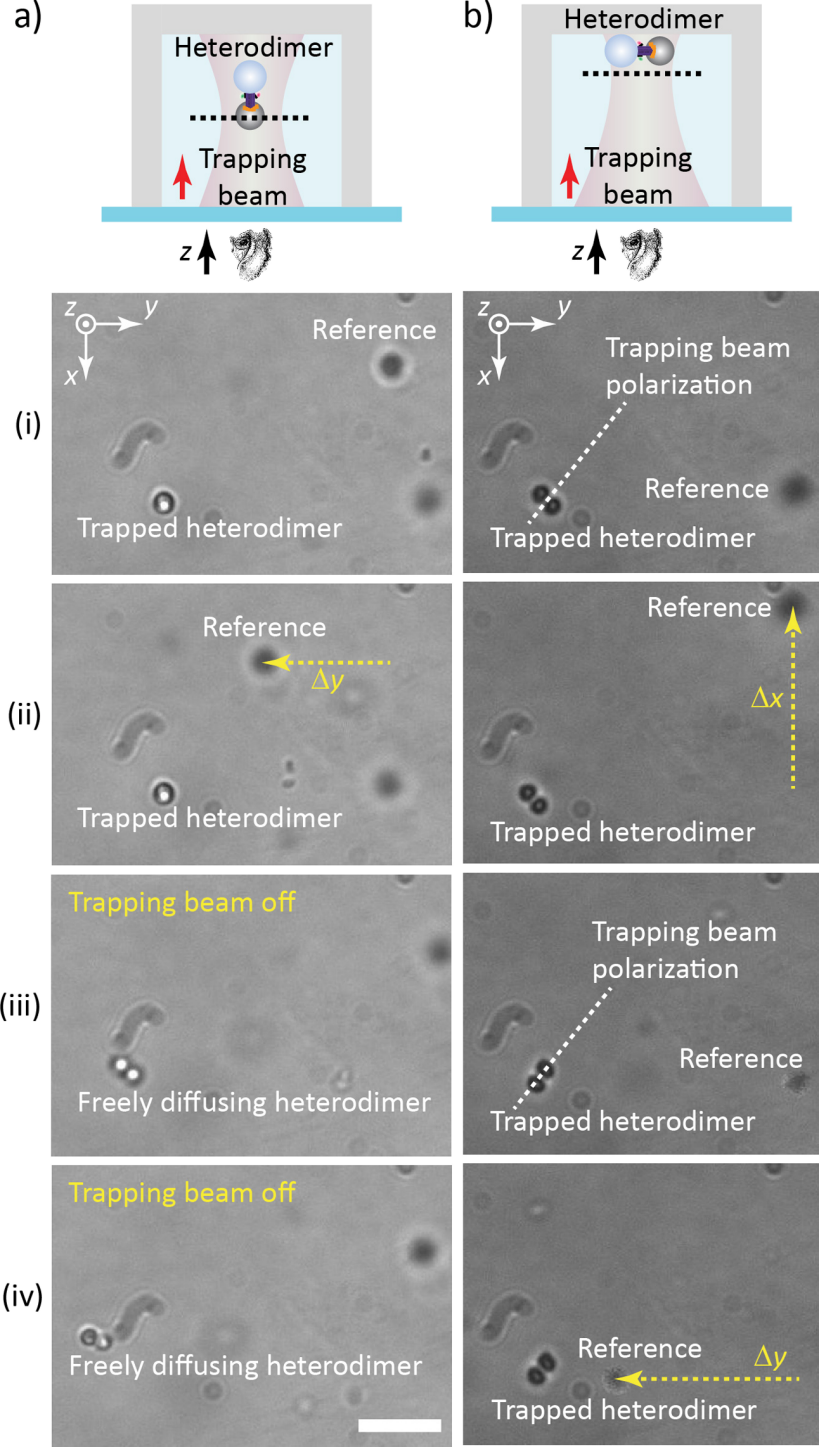
Optical trapping and manipulation of colloidal heterodimers in different trapping geometries. a) Full 3D optical manipulation in the bulk liquid. b) 2D surface-assisted optical manipulation in the vicinity of the sample chamber wall. Panels (i)–(iv) illustrate various phases of the manipulation process (see the main text for the full explanation). Scale bar: 5 μm. In both experiments, which were carried out with two different heterodimers from the same synthesis batch suspended in the same microfluidic chip, trapping beam power was set to ∼35 mW at the sample plane. Dashed lines in the schematic drawings of the sample chamber shown at the top indicate the position of the focal plane of the trapping beam (individual dimensions and distances are not drawn to scale). The individual frames presented in the figure were extracted from supporting videos “3D Dimer Trapping” for panel a) and supporting video “2D Dimer Trapping” for panel b).

Figure 4b demonstrates the 2D surface-assisted manipulation of a heterodimer using the same setup as in part a); the only difference is in the distance of the focal plane of the trapping beam from the top wall of the sample chamber. Confinement of the heterodimer to the proximity of the sample chamber surface restricted the configuration space of the heterodimer, which could not freely rotate to orient itself along the axis of the trapping beam to reach the global minimum of the interaction energy with the optical field. Consequently, the heterodimer aligned itself along the direction corresponding to the local minimum of the interaction energy, which is tied to the trapping beam polarization.^6^ The actual orientation of the heterodimer with respect to the beam polarization direction was then determined by the interplay between the anisotropic polarizability and dimensions of the heterodimer and the local intensity profile of the trapping beam.^38^ As indicated in Figure 4b, during the manipulation induced by translating the sample chamber past the stationary optical trap, the heterodimer displayed bistable behavior; it oriented itself either normal to [panels (i) and (ii)] or along [panels (iii) and (iv)] the trapping beam polarization. A histogram of all heterodimer orientations from this particular experiment is provided in Figure S21. Individual panels of Figure 4b directly illustrate that the heterodimer in both spatial orientations remained stably trapped during translations along the *x*-axis [panels (i) → (ii)] and along the *y*-axis [panels (iii) → (iv)].

During the optical manipulation, trapped heterodimers were held in a focused infrared trapping beam with the power at the specimen plane reaching tens of mW. As discussed in^37^, magnetic particles in the heterodimers can experience heating due to the absorption of the trapping light, which could potentially have a negative impact both on the structural integrity of the heterodimers and on the intensity of fluorescence emission from the dye molecules attached to the 24HB. However, the observation of the trapped heterodimers confined in 2D using the power of ∼30 mW at the sample plane did not indicate any detectable disintegration due to optically induced heating for up to 10 min of continuous infrared illumination. In addition, we also tested the stability of fluorescence emission from the trapped heterodimers. As shown in Figure S22, fluorescence signal from the heterodimer did not display any appreciable decay after more than 20 s of continuous optical confinement, further confirming the robustness of our optomechanical probes.

In conclusion, we successfully demonstrated the use of DNA origami for the synthesis of composite heterodimers with well-defined structural and material properties that can serve as custom probes for optomechanical experiments. To this end, we used the 24HB DNA origami scaffold with localized binding sites for the attachment of fluorescent dyes and for the selective coupling to microspheres with distinct surface chemistries (streptavidin vs azide) and material characteristics (polystyrene vs Magnefy). TEM and FM imaging of the assembled heterodimers confirmed that they were indeed formed by the selective 24HB linking, and the emission from the fluorescently labeled 24HB was not impaired by its coupling to the microspheres. The synthesis procedures were optimized, reaching heterodimer yields of ∼15% . Our results show that DNA origami can directly guide the self-assembly of the desired microscopic structures, without using additional solid templates aiding the synthesis.

Optical tweezers were used to manipulate the heterodimers suspended in aqueous buffers within microfluidic chips. Due to their shape and material asymmetry, the heterodimers could be stably confined in different orientations with respect to the trapping beam. The optical trapping experiments confirmed the mechanical integrity and steady fluorescence signal of the probes after their prolonged exposure to the focused trapping beam and to the hydrodynamic forces exerted by the flowing suspension liquid. Systematic characterization of the response of the probes to trapping light with controlled polarization state (linear, circular) and transverse profile of optical intensity, and utilization of these optically driven rotations for environmental sensing are currently underway.

The presented nanofabrication approach based on selective binding of microspheres of different materials via DNA origami linkers allows for straightforward modifications of the size and material of the constituent particles for the specific intended applications such as optically- and magnetically induced directional transport for targeted drug delivery and biomedical nanorobotics^1,2,39^ and patchy-particle^15,40^ or field-assisted^41^ metamaterial self-assembly.

Apart from serving as a selective linker for microparticles, DONs provide a programmable platform for the controlled attachment of a variety of functional components such as dye molecules, quantum dots, or metallic nanoparticles via well-established methods.^42,43^ Consequently, they enable the creation of nanoscale guides for directional energy transport,^44,45^ plasmonic structures,^46^ unidirectional emitters,^47^ and possibly even exciton-based quantum logical gates^48^ that can be integrated with optically trapped probes to further extend their potential for quantitative sensing of various environmental characteristics.

Our TEM images demonstrate the stability of the heterodimers under vacuum conditions when deposited on the surface of a TEM grid, making the system promising for experimental studies in vacuum. In combination with complex optical architectures enabled by the DONs,^44–48^ this observation paves the way for novel quantum optomechanics^49,50^ and quantum gravity^51,52^ experiments.

## Supplementary Material









## Data Availability

The raw data set that supports the findings of this study is available at DOI:10.5281/zenodo.18044958

## Notes

Detailed description of experimental procedures including DNA origami design, DNA origami synthesis, fluorophore attachment, fluorescence microscopy, microsphere hybridization, atomic force microscopy (AFM), transmission electron microscopy (TEM), optical trapping of hybrid colloidal heterodimers, magnetic actuation of heterodimer orientation, DNA nanostructure sequence details including CaDNAno design, left linker strand sequences for binding of polystyrene microspheres, right linker strand sequences for binding of magnetic microspheres, extended staples for Cy3, extended staples for 6-FAM, core staples, supplementary figures including AFM images of the 24HB without extensions, AFM images of the 24HB extended with Cy3 and 6-FAM dyes, fluorescence microscopy (FM) images, TEM images, histogram on bistability of heterodimers optically trapped in 2D, and images showing the effects of prolonged exposure to the trapping light on the fluorescence emission of optically trapped heterodimers (PDF)
Magnetic manipulation (AVI)
2D Dimer Trapping (MOV)
3D Dimer Trapping (MOV)
