# Supplementary material for: Optomechanical Probes with Tailored Material and Shape Asymmetry Assembled Using DNA Origami
Source: Nano Lett. 2026 Jan 21;26(6):2080–8. doi: 10.1021/acs.nanolett.5c05354 (PMC12922178; doi:10.1021/acs.nanolett.5c05354)
Supplement: Supplementary file 1 [file nl5c05354_si_001.pdf]

# Supporting Information: Optomechanical Probes with Tailored Material and Shape Asymmetry Assembled Using DNA Origami

David Daniel Ruiz Arce,[†] Markéta Benešová,[‡,¶] Václav Protiva,[†] Jaroslav Kočišek,[†] Zdeněk Pilát,[‡] Jan Ježek,[‡] Lukáš Šilhan,[‡] Pavel Zemánek,[‡] Alexandr Jonáš,[*,‡] and Leo Sala[*,†]

†*Department of Dynamics of Molecules and Clusters, J. Heyrovský Institute of Physical Chemistry of the Czech Academy of Sciences, Dolejškova 3, 18200 Prague, Czech Republic*

‡*Department of Microphotonics, Institute of Scientific Instruments of the Czech Academy of Sciences, Královopolská 147, 61200 Brno, Czech Republic*

¶*Faculty of Chemistry, Brno University of Technology, Purkyňova 118, 612 00 Brno, Czech Republic*

E-mail: sasa@isibrno.cz; leo.sala@jh-inst.cas.cz

# Contents

**1 Experimental Section** **S4**

1.1 DNA origami design . . . S4

1.2 DNA origami synthesis . . . S4

1.3 Fluorophore attachment . . . S5

1.4 Fluorescence microscopy . . . S5

1.5 Microsphere hybridization . . . S6

1.6 Atomic force microscopy (AFM) . . . S6

1.7 Transmission electron microscopy (TEM) . . . S7

1.8 Optical trapping of hybrid colloidal heterodimers . . . S7

1.9 Magnetic actuation of heterodimer orientation . . . S10

**2 Design and sequence details** **S11**

2.1 CaDNAno design . . . S11

2.2 Left linker strand sequences for binding of polystyrene microspheres . . . S12

2.3 Right linker strand sequences for binding of magnetic microspheres . . . S13

2.4 Extended staples for Cy3 . . . S14

2.5 Extended staples for 6-FAM . . . S15

2.6 Core staples . . . S16

**3 Supplementary figures** **S20**

3.1 AFM images of the 24HB without extensions . . . S20

3.2 AFM images of the 24HB extended with Cy3 and 6-FAM dyes . . . S21

3.3 Fluorescence microscopy (FM) images . . . S22

3.4 TEM images . . . S31

3.5 Bistability of heterodimers optically trapped in 2D. . . . S33

3.6 Effects of prolonged exposure to the trapping light on the fluorescence emission of optically trapped heterodimers . . . S34

**References** **S34**

# 1 Experimental Section

## 1.1 DNA origami design

As a starting point, we used the 24HB DNA origami structure reported by Kuzyk et al.,[1] which was modified using caDNAno v.0.2.5 (See Figure S3). Specifically, to conjugate the 24HB with the microspheres, 14 staples located at each end of the structure were extended at either the 5' or the 3' end (depending on their respective outermost termini) with thymine oligonucleotides. These linker strands were then functionalized either with biotin at the L-end (polystyrene microsphere link) or with DBCO at the R-end (Magnefy™microsphere link). Furthermore, we carefully selected and extended 45 staples, distributed uniformly along the 24HB structure, with 20 nt overhangs to serve as binding sites for 2 different fluorophores attached to ssDNA strands that were complementary to their target binding sites (see Section 1.3 for additional details). The mechanical and structural stability of the modified 24HB DNA origami was analyzed using the CanDo software[2] (see Figure S4), showing no distortions or global twists due to the modifications.

## 1.2 DNA origami synthesis

The 24HB structures were assembled by annealing the p7560 scaffold (Tilibit Nanoystems) at a final concentration of 20 nM with 5× excess of the standard staple strands and 10× excess of the extended or modified staples (Metabion) in a folding buffer (FOB) containing 1× TAE and 20 mM $MgCl_2$. The annealing process was carried out in a heating block (BioSan CH-100) modified with an Arduino platform to precisely control the temperature ramping. The annealing protocol began with a temperature increase from 23 °C to 90 °C for 12 min, followed by gradual cooling to 85 °C for 5 min. The temperature was then decreased to 65 °C over a span of 1 h, reduced to 44 °C over a span of 30 h, and subsequently lowered to room temperature for 18 h. Afterwards, the folded structure was filtered by performing three exchanges of 1× FOB in an Amicon® Ultra-0.5 centrifugal filter (100kDa MWCO,

Millipore) to remove the non-hybridized staples. The final concentration of the assembled 24HB was measured using a Denovix DS-11 FX+ microvolume UV-Vis spectrometer.

## 1.3 Fluorophore attachment

We labeled the 24HB DNA origami with fluorophores linked to ssDNA oligomers that were hybridized to complementary binding sites positioned near the origami surface. Specifically, 26 fluorophore binding sites were extended with 20-nt for Cy3 labeling and 19 binding sites were extended with 20-nt overhangs for 6-FAM labeling (see SI Tables S3 and S4). This strategy enabled precise, site-specific dye incorporation close to the origami framework while minimizing nonspecific fluorophore interactions, thereby offering high versatility in fluorophore placement. Fluorophore incorporation occurred during the annealing process, and unbound strands were effectively removed through centrifugal filtration.

## 1.4 Fluorescence microscopy

The fluorescently labeled 24HB and the hybrid structures "microspheres - 24HB" were immobilized on a chambered coverslip ($\mu$-Slide 8 well, uncoated; ibidi, Cat. No. 80821). The samples were incubated in 300 $\mu$L of 1× TAE buffer supplemented with 20 mM $MgCl_2$ for 1 hour. Fluorescence microscopy was performed using an inverted microscope (IX73; Olympus) equipped with a TIRFM oil-immersion objective (PlanApo 100×/1.45 Oil; Olympus) and a cooled LED light source (pE-4000; CoolLed). Fluorescence detection was carried out using the following filter sets: U-FRFP (Ex. 535–555 nm, Em. 570–625 nm, dichroic mirror (DM) 565 nm) for Cy3, U-FGFP (Ex. 460–480 nm, Em. 495–540 nm, DM 490 nm) for 6-FAM, and U-FUNA (Ex. 360–370 nm, Em. 420–460 nm, DM 410 nm) for UV. Images were captured using a cooled CMOS camera (ASI 294MM Pro; ZWO) controlled via ASICap software. All images were acquired at room temperature (RT) with the excitation light intensity set to 50% of the maximum and the exposure time of 500 ms for all channels. Image analysis and processing were performed using Fiji (ImageJ) software.

## 1.5 Microsphere hybridization

In this work, we employed two different types of microspheres: streptavidin-coated polystyrene microspheres and azide-coated click Magnefy™, microspheres, both commercially available (Bangs Laboratories, Cat. No. CP01004 and CBMFY01a, respectively). For a typical experiment, 10-20 $\mu$L of the beads were washed four times with ten-times their volume of the bead coupling buffer (pH 7.4, Bangs Laboratories, Cat. No. BUFF3) by centrifugation at 5000 rpm for 5 min. The pellet was re-suspended in 10-20 $\mu$L of 24HB solution in the folding buffer with the concentration of 0.026, 0.26, and 2.6 nM, corresponding to microsphere-to-24HB ratios of 1:1:1, 1:1:10 and 1:1:100. To test the specificity of conjugation of the 24HB to the two types of microspheres, each microsphere type was conjugated separately by mixing either the azide-coated or streptavidin-coated microspheres with the 24HB tagged by Strain-Promoted Alkyne-Azide Cycloaddition (SPAAC) with DBCO and biotin at its ends, respectively (see Section 4.1 for details). Mixtures were incubated overnight at RT with gentle mixing (400 rpm) in a shaker. Unbound DNA was removed by two additional washes through centrifugation at 5000 rpm for 1 min. Finally, the hybrid complexes were re-suspended in 10 $\mu$L of the folding buffer and diluted as required by the subsequent experiments.

## 1.6 Atomic force microscopy (AFM)

To verify the shape of the assembled 24HB origami structures by AFM, they were deposited onto 12 mm mica discs (grade v1, NANOandMore, Cat. No. 50-D-12). Initially, the first few monolayers of mica were removed using an adhesive tape to obtain a clean surface. Then, 20 $\mu$L of a 2 nM sample was dropped onto the fresh surface and left to incubate for 30 min before washing it off with MilliQ water and drying with $N_2$. All dried samples were imaged on Dimension Icon AFM (Bruker) in ScanAsyst-Air tapping mode using SCANASYST-AIR probes (tip radius  2 nm, resonance frequency 70 kHz, spring constant 0.4 N/m). All images were acquired at a resolution of 512 $\times$ 512 pixels, with a scan rate of 1 Hz and different scan sizes. Image analysis was conducted using Gwyddion software.

## 1.7 Transmission electron microscopy (TEM)

For the TEM characterization of the 24HB and of the fully assembled heterodimer microstructures, 5 $\mu$L of 2 nM 24HB solution or a 20× diluted solution of heterodimer microstructures was deposited onto a TEM grid with a continuous ultrathin carbon film surface on top of lacey carbon supported by a 300 mesh copper grid (Sigma Aldrich, Cat No. 930326-1EA) and allowed to incubate for 5 min. The sample was then stained with 2.5 $\mu$L of a 1% uranyl formate solution for 5 min, after which the droplet was removed with a filter paper. Excess stain was carefully removed by a subsequent wash using 30 $\mu$L of MilliQ water. The grid was then allowed to dry for at least 3 h before imaging. All TEM imaging was performed in a Jeol JEM2100Plus transmission electron microscope operated at 200 kV, enabling high-resolution visualization of the individual DNA nanostructures and the heterodimer structures.

## 1.8 Optical trapping of hybrid colloidal heterodimers

Optical trapping and manipulation of the synthesized colloidal heterodimers was implemented by attaching a compact custom-made fiber-optical module to a standard epi-fluorescence microscope (Olympus IX 70; see the schematic drawing and the picture of the fiber-optical module in Figure S1a). Briefly, trapping light from a laser diode (wavelength 1064 nm, maximal output power 250 mW) was coupled into a single-mode optical patch cable connected to the module that contained an adjustable collimator lens and an adjustable dichroic mirror (DM2) reflective at the infrared trapping wavelength and transparent within the visible part of the spectrum (~400 - 950 nm). The module was then inserted between a microscope objective with a high numerical aperture (PlanC N 100x, NA = 1.25, oil immersion; Olympus) and revolving objective holder of the microscope (see the inset in Figure S1a), redirecting the trapping light along the optical axis of the microscope. The used objective transmitted approximately 40 % of the infrared trapping light to the sample plane. Since DM2 displayed high transmission in the visible spectral region, it allowed fluorescence exci-

tation and observation of emission from optically trapped heterodimers on a high-sensitivity, thermoelectrically cooled CMOS camera (ASI294MM Pro; ZWO) using the standard epi-fluorescence arrangement (excitation and emission filters Ex and Em, dichroic beamsplitter DM1), with a high-pressure Hg lamp serving as the excitation source. To prevent the trapping light reflected from the sample chamber walls from reaching the camera, a short-pass filter F blocking the light with wavelength above 995 nm (FESH1000; Thorlabs) was placed in front of the camera.

In the configuration described above, the objective served both for carrying out fluorescence microscopy and for focusing the trapping beam to a diffraction limited spot to create a single-beam optical trap (optical tweezers). Adjustments of the collimator lens and DM2 then enabled three-dimensional (3D) positioning of optically trapped heterodimers with respect to the optical axis and the focal plane of the microscope. In addition to the trap positioning, the whole sample could also be independently moved relative to the optical trap using the 3D positioning stage of the microscope. Hence, it was possible to fully control the location of the trapped heterodimers both within the field of view and with respect to the sample chamber.

For the trapping experiments, optically manipulated heterodimers were suspended in an aqueous working buffer (1× TAE + 20 mM $MgCl_2$ + 0.01% TWEEN 20) within custom-designed microfluidic chips whose flow channels, featuring liquid reservoirs at both ends, were formed by interconnected rhomboidal chambers (see Figure S1b). The reservoirs were connected to metal electrodes that allowed applying DC voltage with adjustable amplitude and polarity between the opposite ends of the channel, inducing electro-osmotic flow with controlled speed and direction along the channel. The particular used shape of the flow channels with a periodically varying channel width then allowed hydrodynamic focusing of the objects suspended in the working buffer on the channel axis. In addition, intermittent low-speed electro-osmotic flow was also used to prevent adhesion of the suspended objects to the surface of the chip. In the experiments, the flow was driven using a home-built DC

voltage source with switchable output polarity and maximal output voltage of 370 V.

The chips were fabricated from polydimethylsiloxane (PDMS) using the standard soft-lithography protocol. After the PDMS curing step, the actual flow channels with the height of $\sim 50\,\mu$m were formed by attaching the PDMS block to a regular glass coverslip for optical microscopy. This coverslip, which was irreversibly bonded to the bottom surface of the PDMS block, both sealed the channel and enabled optical access to the channel using the inverted optical microscope configuration. The studied samples were loaded into the flow channels by depositing a droplet of heterodimer solution with the desired concentration into one of the two channel-end reservoirs and subsequently activating unidirectional electro-osmotic flow in the direction away from the input reservoir. During the optical manipulation experiments, the flow was stopped to minimize disturbances of the trapped objects by hydrodynamic forces.

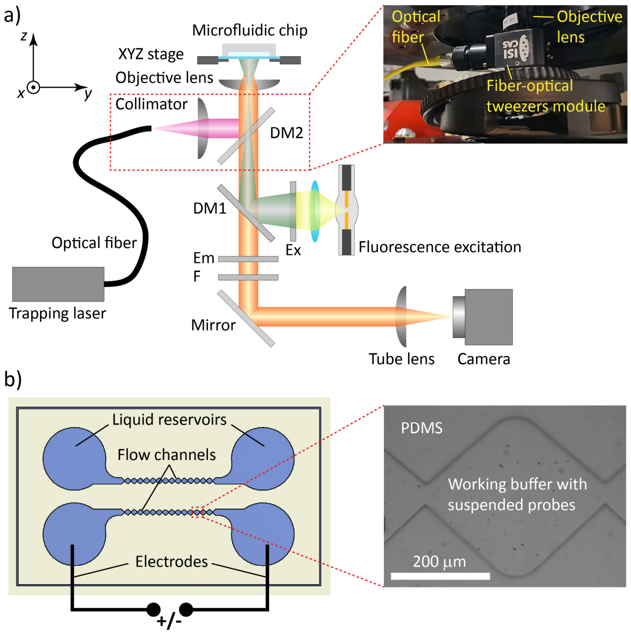


Figure S1: a) Experimental setup for simultaneous optical trapping and fluorescence microscopy of fluorescently labeled heterodimers. Inset shows the module of fiber-optical tweezers attached to the microscope. DM1, DM2 - dichroic mirrors, Em - emission filter, Ex - excitation filter, F - cut-off filter for the trapping light wavelength. b) Microfluidic chip with the capacity of inducing electro-osmotic flow used in the reported optical trapping experiments. Left: Overall layout of the chip, right: detail of the actual fabricated flow channel filled with a suspension of fluorescently labeled heterodimers in an aqueous working buffer.

## 1.9 Magnetic actuation of heterodimer orientation

Figure S2 provides a schematic illustration of the procedure used in the experiments with magnetic actuation of the synthesized heterodimers. The heterodimers suspended in the working buffer were injected into a sealed sample chamber formed by two microscopy cover glasses whose separation distance was adjusted by 30$\mu$m spacer beads. Subsequently, the heterodimers were exposed to a varying external magnetic field of a permanent magnet that was moved across the sample chamber in an oscillatory fashion. Simultaneously with the motion of the magnet, the beads were observed through the microscope in the bright-field imaging mode (see also Figure S1) and their response was recorded. We concentrated on analyzing the response of heterodimers and larger clusters of microspheres that were loosely adherent to the surface of the sample chamber. Using this approach, the center-of-mass motion of the studied objects was prevented, and the response was restricted to changes in the orientation of the object, which were easier to track over extended time periods. See the supporting video "Magnetic Manipulation" for an example.

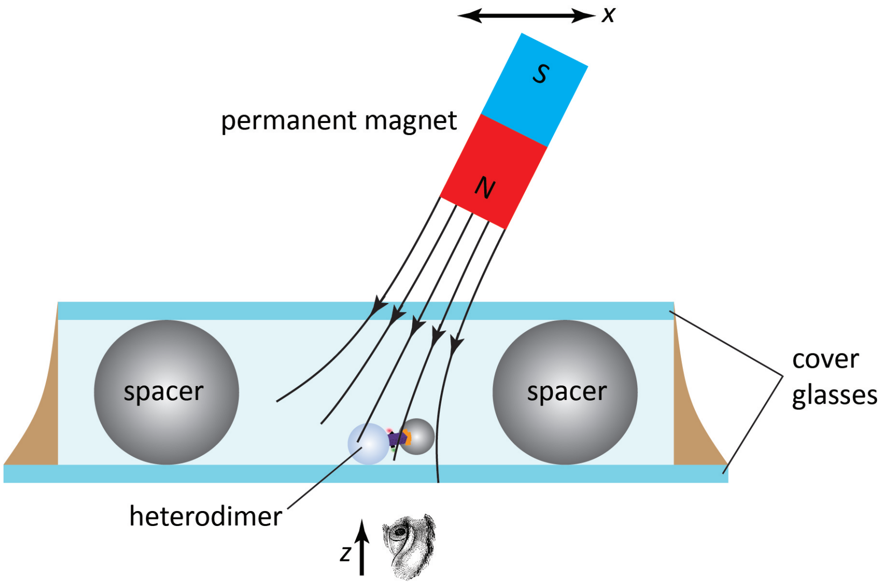


Figure S2: Schematic illustration of magnetic actuation of heterodimers suspended in the working buffer. The heterodimers are exposed to the varying magnetic field of a permanent magnet moving along the x-axis and simultaneously observed along the z-axis. The spacers, heterodimer, and magnet are not drawn to scale.

# 2 Design and sequence details

## 2.1 CaDNAno design

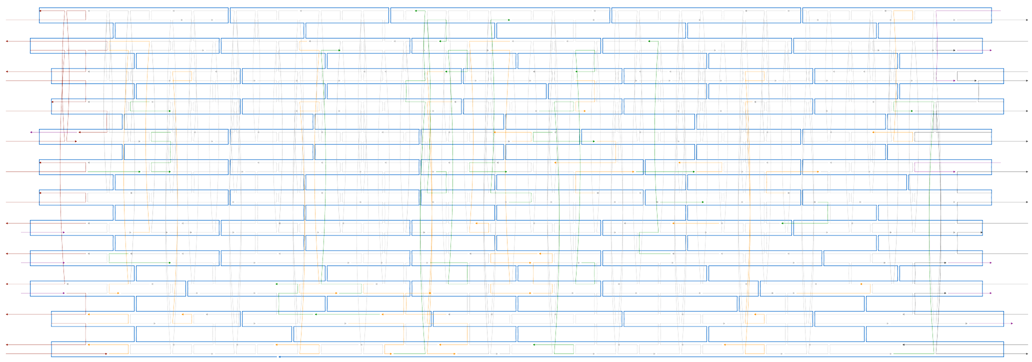

Figure S3: CaDNAno design diagram of the 24HB. Blue: scaffold; grey: core staples; orange: Cy3 binding sites; green: 6-FAM binding sites; red: left linker strands with biotin; black: right linker strands with DBCO; pink and purple: left and right poly-T blockers, respectively.

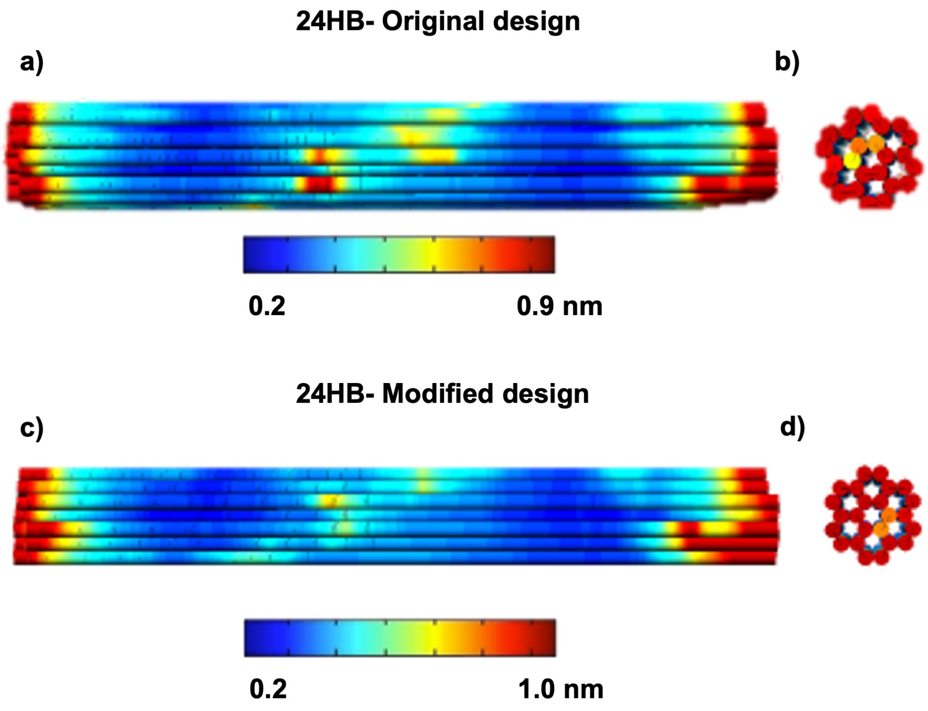


Figure S4: CanDo simulations of the root-mean-square fluctuations (RMSF) of the 24HB. The heat map blue and red represent the low and high relative flexibility, respectively. a) and c) top; b) and d) side view.

## 2.2 Left linker strand sequences for binding of polystyrene microspheres

Table S1: Table of position names and sequences of the left linker strands.

| Position name | Sequence |
|---|---|
| L-3[2]-18[0] | AAAGGCTATCAGCTTTCCGGCACC-TTTTTTTT-Biotin |
| L-19[14]-20[1] | GGAAACCAACTGTTGGGAA-TTTTTTTTTTTTTTTTTT-Biotin |
| L-1[0]-9[17] | Biotin-TTTTTTTTTTTTTT-GTTCTAGCTGATAAATAATATGAATTA |
| L-11[0]-10[5] | Biotin-TTTTTTTTTTTTTT-ATCAAAAATAATTCGCAACCAATAGGAACGCC |
| L-21[9]-22[1] | GGGCGATCGGTGAGGGGGATGTGC-TTTTTTTTTTTTTTTTTT-Biotin |
| L-5[1]-6[9] | Biotin-TTTTTTTTTTTTTTTTTT-TAAAACTAGCATAAAGCCCCAAAA |
| L-14[20]-14[0] | CGTGGGAACAAACGGCGGA-TTTTTTTT-Biotin |
| L-3[14]-4[1] | GTCATTGGAACGGTAATCG-TTTTTTTTTTTTTTTTTT-Biotin |
| L-3[21]-2[0] | CCTGAGAAGGGTAGCTATTTTTGAGAGATCTAC-TTTTTTTT-Biotin |
| L-13[0]-12[5] | Biotin-TTTTTTTTTTTTTT-TAAATGTGAGCGAGTACAGCTTTCATCAACAT |
| L-16[20]-16[0] | GCGCATCGTAACCGTGCAT-TTTTTTTT-Biotin |
| L-23[1]-23[27] | Biotin-TTTTTTTTTTTTTTTTTT-TGCAAGGCGATTAAGTTGG |
| L-9[0]-0[5] | Biotin-TTTTTTTTTTTTTT-AAAATTCGCTATTCAACC |
| L-7[1]-8[18] | Biotin-TTTTTTTTTTTTTTTTTT-ACAGGAAGATTGTATAAGCATTGTAAACG |
| L-19[0]-19[13] | TTT-GCTTCTGGTGCC |
| L-17[0]-17[13] | TTT-CTGCCAGTTTGA |
| L-8[17]-8[2] | TTAATATTTTGTT-TTT |
| L-15[0]-15[13] | TTT-TTGACCGTAATG |

## 2.3 Right linker strand sequences for binding of magnetic microspheres

Table S2: Position name and sequences of the right linker strands.

| Position name | Sequence |
|---|---|
| R-6[314]-23[331] | CTCCAACGTCAAAGGCCTTGAATCGGCTGACGCATTT-TTTTTTTTTTT-DBCO |
| R-16[331]-15[316] | DBCO-TTTTTTTTTTTTTTT-GCTAACTCACCAGCTGCA |
| R-1[308]-1[331] | CGAAAGGAGCGG-TTTTTTTTTTT-DBCO |
| R-6[323]-5[331] | GAACGTGGAGAACCATCATTTTTTT-DBCO |
| R-12[319]-11[331] | TGAGACGGGCAAAGAGTTGCAGCATTTTTTTTTTTT-DBCO |
| R-18[331]-17[304] | DBCO-TTTTTTTTTTTTTTTACACAACATACGAGCCGGAAGCAAGCCTGGGGTG |
| R-7[294]-9[331] | TCCAGTTAGCCCGAATGGTGGTTCCGAAATCGGCAAAATCTTTTTTTTTTTT-DBCO |
| R-22[331]-22[290] | DBCO-TTTTTTTTCACATAAATCATTTCTCCGAACTCTGACCTCCTG |
| R-8[319]-7[331] | CCTTATAAATCAAAAGAATTGGAACAAGAGTCCACTATTAAATTTTTTTT-DBCO |
| R-4[331]-5[314] | DBCO-TTTTTTTTCCCAAATCAAGTTTTTACTACGT |
| R-14[331]-13[331] | DBCO-TTTTTTTTTTTTTTTTTAATGAATT |
| R-14[331]-13[331]-2 | CTTTTCACCAGTTTTTTTTTTTT-DBCO |
| R-20[331]-19[304] | DBCO-TTTTTTTTCGGAGGATCCCCGGGTACCGAGCTCGAATTAATTGTTATCC |
| R-2[331]-3[307] | DBCO-TTTTTTTTTTTTTTTCCCCGATTTAGAGCTTGAACCCT |
| R-0[322]-5[307] | TTTGCGCTAGGGCGCTGGCAAGATGGCCC |
| R-17[305]-17[319] | CCTAATGAGTGATTT |
| R-21[312]-21[326] | CCTCGATAAAGATTT |
| R-10[322]-11[307] | TTTAGCGGTCCACGCTGGTTTGCCCCAGCCGCCTGGCCCTGAG |
| R-3[308]-3[319] | AAAGGGAGCTTT |
| R-19[305]-19[319] | GCTCACAATTCCTTT |

## 2.4 Extended staples for Cy3

Table S3: Sequences of the binding sites staples for Cy3.

| Position name | Sequence |
|---|---|
| ext-Cy3-11[222] | TGATTATCATAATGGAAGACTAGAGCTTCGCTCCGAAATAAAAT |
| ext-Cy3-2[160] | TAAGTATTTGGGAAGATTGGCCCAGAGCTTCGCTCCGAAATAAAAT |
| ext-Cy3-5[182] | AAAAGAAGAAACGCACAAGCTTCGCTCCGAAATAAAAT |
| ext-Cy3-19[168] | TATTCTATTGCGGGGCCTTAACAATTGCTTCGCTCCGAAATAAAAT |
| ext-Cy3-18[115] | CCGCGCCACCCTCAGAACGGAATAGGGCTTCGCTCCGAAATAAAAT |
| ext-Cy3-4[138] | GGTAGCAAAACAGTTAATGCACATGAAAGTGCTTCGCTCCGAAATAAAAT |
| ext-Cy3-9[161] | AGCAAGAGCCCTTTTTCGGCGCTTCGCTCCGAAATAAAAT |
| ext-Cy3-1[287] | CGAGAAAGTCACGCCTGTTTGGATAGGGTTTAACCGCTTCGCTCCGAAATAAAAT |
| ext-Cy3-9[189] | CGTTAAAGAGATAACCCACAAGAATTGAGCTTCGCTCCGAAATAAAAT |
| ext-Cy3-12[251] | AAACCACATCAATATAATCTATCCAGCTTCGCTCCGAAATAAAAT |
| ext-Cy3-12[188] | GAATAACAAGTCAGAGGGTAATTGAGCGCTTCGCTCCGAAATAAAAT |
| ext-Cy3-14[229] | CGAACGTTAATTAAACGCGCTTCGCTCCGAAATAAAAT |
| ext-Cy3-16[169] | TTATCCTGAATCTTTGCTGAATTTATTGAAGCTTCGCTCCGAAATAAAAT |
| ext-Cy3-19[255] | CATCGCCATTAAAAATACCGAACGCATTTGCTTCGCTCCGAAATAAAAT |
| ext-Cy3-15[36] | CGAACCTTTTAGAACCCAGGTCTGGAGGAAGATGCGCGCTTCGCTCCGAAATAAAAT |
| ext-Cy3-2[93] | TAAGACGGTCGCAGATAAAACCAAAAAGGTGCTTCGCTCCGAAATAAAAT |
| ext-Cy3-20[139] | GTATAGTATAGCCCCGTACAGGAGCTTCGCTCCGAAATAAAAT |
| ext-Cy3-3[154] | CCGGAAAGTTCCAGTAACGGGGTTCGGGCTTCGCTCCGAAATAAAAT |
| ext-Cy3-5[238] | TTACAAACCTGAGCAGCATCACAATGCTTCGCTCCGAAATAAAAT |
| ext-Cy3-5[49] | TTAAGCAGTACCAACCTCAAAATAAGCTTCGCTCCGAAATAAAAT |
| ext-Cy3-21[21] | CGGGCCTCTTCGCTCATTCAAGCTGCGCGCTTCGCTCCGAAATAAAAT |
| ext-Cy3-6[240] | ATTTATGATAGCTGAGAGCCAGCAGCTTCGCTCCGAAATAAAAT |
| ext-Cy3-7[91] | AACCGGCAGGCGCAGAGAAGCAGCGGGGTTGTAAAAGCTTCGCTCCGAAATAAAAT |
| ext-Cy3-23[28] | GTAACGCCGCCAGCTGGCGAACTTCGCTCCGAAATAAAAT |
| ext-Cy3-21[107] | ATAAGTGCCGTCGAGAGGGGAGACTCTGGGCTTCGCTCCGAAATAAAAT |
| ext-Cy3-6[141] | ACCGATATATGCGCACGACGCTTCGCTCCGAAATAAAAT |
| ext-Cy3-8[154]-9[160] | ATTTCGGTCATAATCAAAAA |
| ext-Cy3-11[263]-12[252] | GAAATTTTGACGCAAAG |
| ext-Cy3-12[202]-12[189] | AGCCTTTACAGAGA |
| ext-Cy3-14[213]-14[198] | TCAACAGTAGGGCTTA |
| ext-Cy3-15[98]-2[94] | CCAGTACAGGCACCAACC |
| ext-Cy3-19[135]-20[140] | GTGTACTGGTAAGTGCCC |
| ext-Cy3-10[244]-0[238] | GATTCTCACATAAATATAAGATTATACATCAAAATGTAAAT |
| ext-Cy3-8[244]-6[241] | AAGAAATTATACAGACGG |

## 2.5 Extended staples for 6-FAM

Table S4: Sequences of the binding sites staples for 6-FAM.

| Position name | Sequence |
|---|---|
| ext-6FAM-34[123] | GATTCTCACATAAATATAAGTTAGTACCCACCCTCAGAGCATAATTTCA |
| ext-6FAM-30[179] | GATTCTCACATAAATATAAGTCCAAATAGCGCATTAGACGAGCCTTATT |
| ext-6FAM-24[141] | GATTCTCACATAAATATAAGTTGTGAATTCATTCGGTCCAGCGAAGGAA |
| ext-6FAM-32[203] | GATTCTCACATAAATATAAGAGTTGGCGAACCGGTGGCACTTATTA |
| ext-6FAM-25[155] | GATTCTCACATAAATATAAGTACCGAAAACAATGAAATAGCAATAAGG |
| ext-6FAM-28[219] | GATTCTCACATAAATATAAGGCTTAGTATCATATGCGTCC |
| ext-6FAM-6[41] | GATTCTCACATAAATATAAGGCAAACCGCTGAAAAGGTGGC |
| ext-6FAM-8[48] | GATTCTCACATAAATATAAGTTTTCATCCATTAGCTGCGAAACGGTGT |
| ext-6FAM-7[170] | GATTCTCACATAAATATAAGGCAGATAGCCGAAATAATAATGACAAACTCATCGAGAACA |
| ext-6FAM-10[155] | GATTCTCACATAAATATAAGCCGGAACCAGCCACCCTCGGAG |
| ext-6FAM-11[136] | GATTCTCACATAAATATAAGCCCTGAGCCACTATCACCCAG |
| ext-6FAM-11[21] | GATTCTCACATAAATATAAGGTCTGGCCTTGCAACTAA |
| ext-6FAM-13[262] | GATTCTCACATAAATATAAGTCGTCAGTCACACGACACAA |
| ext-6FAM-13[210] | GATTCTCACATAAATATAAGTATAAAGCCAATTTT |
| ext-6FAM-16[213] | GATTCTCACATAAATATAAGAGGCAGAGGCAAACAACGTAGT |
| ext-6FAM-16[34] | GATTCTCACATAAATATAAGGAGCTTGTATCGGCCTATTGCATCAAAA |
| ext-6FAM-17[134] | GATTCTCACATAAATATAAGCAGAATGGAAAGCCATACATTACCATTGGAACGAG |
| ext-6FAM-20[117] | GATTCTCACATAAATATAAGTGTATCACCGTACTCAGGTAGC |
| ext-6FAM-20[95] | GATTCTCACATAAATATAAGGAGAGGCTTTTGACGATAACATAACGC |
| ext-6FAM-23[287] | GATTCTCACATAAATATAAGGCACGAATATAGGGGGCGAAAAACCGTTGTGTTGT |
| ext-6FAM-11[160]-10[156] | AATTAACCCAAATAATATCA |
| ext-6FAM-2[136]-15[132] | CAAAATCACTATCAGGTT |
| ext-6FAM-14[249]-13[261] | CTCGTCATTTTGCGGAACTCAA |
| ext-6FAM-13[196]-13[209] | ATGAAAATAACCAG |
| ext-6FAM-16[229]-16[214] | TAATACATTTGAATTT |
| ext-6FAM-16[51]-16[35] | ATATCGCGTTTTAATTC |

## 2.6 Core staples

Table S5: Table core staples sequences part 1.

| Position name | Sequence |
|---|---|
| stap10[174]-2[179] | GTTAAGCGAACACCCTGAACAATAAAAAAGAAAATAAA |
| stap19[58]-19[76] | CTATCATAACCCTCGTTTA |
| stap19[248]-23[250] | CCTACCTCAATCCTTGCTGTGCCACCCGACA |
| stap5[249]-7[265] | CGCAACAATATAACAGTACCTTTTACAAAGA |
| stap18[167]-14[168] | AGGTTCCCGTAGAAAATACATAAGACACGAGCGTCAACAGCC |
| stap11[112]-18[116] | GAACAACTTGCTAAAATACACTAATGCCGTTTCCAGAA |
| stap5[77]-22[70] | AGGACAGGCTGACCTTCATCAGCCAGTGGACTGGA |
| stap6[279]-13[276] | TCAGTGAAGAATCCAACCACCCGCGTACATGGAAAGGAT |
| stap13[175]-17[183] | ATTTTTTCAAAATATTTCCAGCCAGCTAATCAAGATT |
| stap19[77]-20[60] | CCAGACGCAAAAGAAGTTTGAAAACGAGAAT |
| stap5[21]-0[21] | GTCAATCCACCATC |
| stap20[174]-4[168] | ATAGAAGGCTTACAGGGCCGTTTTTATTTGTTAGC |
| stap14[167]-2[161] | ATATTATTTATCCACTACCAACGCTAACCACGGAA |
| stap8[76]-7[90] | AACGTAAAGCAAGAGTAATCTTGACAAG |
| stap11[73]-15[69] | TGGGGTGAATTGAGATTTGCGACCTGCTCCTAAGAAATC |
| stap23[63]-9[62] | AACGACGTTAACATCTCAGAGAAAAAGATTCAAAATCGCAAA |
| stap10[215]-5[202] | AAAAGCCTGTTAATATCAGATAAGAATAAAACATGATGCTG |
| stap3[229]-14[230] | AACAAACATTCATTTGTATTAGACCTTTGCC |
| stap7[136]-23[156] | CGCCCACGCTAGCGCGTTTTCATAACTTTAGCTAG |
| stap3[105]-14[105] | TGAGGAAACTACGAAAACTACAGTTAGC |
| stap22[136]-3[132] | ATTAAGAGGCTTTGATATAACGGCTGGCAGTA |
| stap20[216]-17[214] | TCCCATCGCCTGTTTCCAGACACCGA |
| stap7[105]-11[111] | CGATAGTGTGAATTCAAAAGGTTGAAAATAGAAAG |
| stap0[128]-11[135] | ATTGGTGAATCACTGTATGGGATTTAAAGGAATTGCCGCCT |
| stap4[266]-21[285] | AGACAGTTAATAAAACAGAGGTGAGGCAGTAAACA |
| stap13[238]-17[254] | ACATTATATTAAATCTTTACAAGAGCCGAAGGTTATCTAAAATAT |
| stap1[63]-11[72] | TAGGTCGACTGCTGAATATAATTTCATTCCATATAAAT |
| stap1[53]-19[57] | GAGGCAAGGATTGCGGGGCAGTCAATTATAACA |
| stap14[197]-13[195] | ATTGAGATGCCAGTTAGTTTAACGTCAAAA |
| stap2[178]-19[181] | CGCAACATAAATCCCGACAGAACGC |
| stap16[307]-17[284] | CATTAATTGCGTTGCGCTCACTGCCCAGATACG |
| stap4[62]-8[56] | ATCGGTTATAAAGCCCAATAACTACTAATAGTAGTCAAATAACCTGTTT |
| stap5[119]-7[135] | CCCTCAGGCTGAGGACAATGACAACAACCAT |
| stap8[55]-22[49] | AGCTATAATCAATTATCATACTCACGACGCGGAAT |
| stap19[21]-18[14] | AGGCAAACGCACTCCAGCCAG |

Table S6: Table core staples sequences part 2.

| Position name | Sequence |
|---|---|
| stap15[196]-1[188] | CATATTTTTTTCGAGCCAGATTTTGCACAGCCTAAATAAAAGTCATATG |
| stap11[280]-0[284] | GCCATTGCAACAACAGGCGAAAAGAACTTAGAATCTGC |
| stap12[34]-9[38] | AAATATCGGCATATATTTTAAGGAGACAAGTT |
| stap5[266]-22[259] | GTACGCCGGCCACCGAGTAATCTGCCATCACCGCC |
| stap4[212]-15[225] | ATATTTTTAAGAGTCAACCAACATGTAGGATTT |
| stap20[59]-23[62] | GACCTGCTTTACAATACTGTTGTAA |
| stap13[225]-1[223] | AAAAGTTTATCATCATATTTAGTAATG |
| stap5[168]-13[174] | GACAGGGGGCGACAAATCAATCAGGGAAAGAAACG |
| stap0[187]-20[175] | CCAAAGACAAAAGAATACCCCGCAGTATTCATCGATCAGAT |
| stap10[265]-10[245] | TGCTGGTAACTGATTGTTTGG |
| stap4[202]-23[208] | GTAAATTGCCATGTAGAAACCCAAGAACCTTCTA |
| stap18[195]-4[203] | ACATGTACTTAATAAGAGAATATAAAGTGACGACAGACTACCACTATAT |
| stap5[150]-13[159] | AGCAGGAGGGAACTTGAGAACCGCCCAGAACCACCCAA |
| stap10[97]-10[98] | AACCCTGACGAGAATGGTTTAATGCGGAGTGAGAATCTCCAA |
| stap17[215]-16[230] | CAAAAGGTAAAAAGGAATTGAGGTCAATAGA |
| stap4[111]-5[104] | GACTAAAGACTTTCGACGCTT |
| stap9[63]-5[76] | TGGTCAAGCTGCTCAAAGTACCAGAAAG |
| stap10[139]-5[149] | CACCGGACGTTTGCTATTGACAGACAGCATCGGATAGC |
| stap22[167]-7[169] | AGCAAAAGAATTGTCAACCTTACAATCAAGTTTGCGAAAAGTAA |
| stap1[161]-5[167] | TTGTCACTTCAACCGATTGAGCCGTAATCAGTAGC |
| stap9[217]-20[217] | TAATTGGAATTTTCGCAAGACGGTTGGGAATCGGCAATAATA |
| stap19[32]-18[42] | CATTCTTTACCCTGACTGAAGCAA |
| stap1[263]-8[266] | CGCTACAGGGACACCCGATCACTTCAATACT |
| stap0[90]-12[98] | ACCAAGCCGCCTGACAACTTTAACTGGCTCAGTTTTGTCGTCTCAACAG |
| stap20[240]-21[231] | ATCTGCTGAACAAGAAATGTGAAAA |
| stap4[276]-14[273] | AAGGGAGCGGGGTTGCTTCTGGCCAATTGGCA |
| stap15[266]-4[267] | GGACATTTGACGAGCACGTATCCTCGTTAGAATCAGATTTT |
| stap11[49]-1[52] | CTGGAAGTGCTGTAGCCT |
| stap15[226]-19[223] | AGAAGAATTACCTTTTTAACCTCCGGAATTCTGTATCAAC |
| stap21[286]-5[293] | GGGCTTAAGCTACGTGAGGTGCCGTAAGGTGTCTAT |
| stap11[39]-10[21] | AGTCGAGTAGATAGCTCATTTTTT |
| stap0[48]-13[69] | AAAGGCCATGCAATGCTCAACTTTTTGCGGATGGCTTAGAGCTTAATCT |
| stap11[238]-18[238] | GCAATTCCAGAAGGATCAATAAACAATTCAAGAAATGGGCAA |
| stap4[167]-20[154] | AAACGTCACCAATGCTATTTCGTGCCTT |
| stap5[294]-1[307] | CAGGGCGTGTAGCGGGAAGGGAAGAAAG |
| stap17[14]-16[21] | GGGGACGACGACAGTAGATGG |
| stap3[283]-20[290] | TAAAAGACAATTGTTTCCTGTGTGACGTA |
| stap16[251]-1[262] | ATAGATTAACAATTTACATTTTATGTGAGTGAAATGCGC |
| stap18[237]-3[228] | ATCAACAGTTGAGTCTTGA |
| stap14[51]-15[35] | AGTACCTTTAATTGCTCCTTTTCGGGTCAAAG |
| stap7[266]-6[280] | GTCTGTCCATCACGCAAATGATTTATAA |
| stap14[272]-10[266] | GATTCACCTGAAATTACCTACCAATATTTCGGCCT |
| stap7[217]-22[217] | CTGACTTAACGCGAACGCTCGTTTCCTT |
| stap8[97]-0[91] | CAGATATTCATTACGAATAAGGCTTGAACGATTAT |
| stap22[195]-18[196] | AAACCAACATTACCAAGACTCACATAGGTCTGAGAATAAACA |

Table S7: Table core staples sequences part 3.

| Position name | Sequence |
|---|---|
| stap1[189]-15[195] | GTTTACGATAGCTTTTTATCAAAATCATTTATCGC |
| stap17[255]-18[261] | CTTTAGGAGACCTGAAAGAATGGCTATTAG |
| stap5[231]-13[237] | TACCAAGCGTCGCTTACATAAAGCGGAATTGAGTA |
| stap14[83]-10[77] | GTCAGGATTTTAAGAATCATTCTTGAGAACACCAG |
| stap22[48]-6[42] | CGTCATAAATATTACAGGGTTTTCCCAGAGGCAAG |
| stap9[18]-4[21] | AATTTTTGTTAAATCTTGTCAAATATATGTAACAAGAGAATCGAT |
| stap16[146]-8[140] | CAAACAAGTCAGACTTAGAGCGTCACCGAGGTAAACATCTTTTCATAGC |
| stap21[232]-5[230] | ATCTAAAAAAGAAGATGATAAATTGAA |
| stap5[203]-22[196] | ATGCAAATTTTCAAATATAGAAACCCCGGGGTATT |
| stap20[51]-21[43] | ATCAAAAATCAGGTCGCATTCATTGA |
| stap15[48]-15[55] | GAAGCAAA |
| stap20[289]-19[254] | ATCATGGTCATAGAACCACCAGCAGAAGAATATCAAACAAA |
| stap9[39]-6[21] | TGATTGGGGCGCTTTAAAAATAGAGGTTGATAATCAGA |
| stap22[289]-23[286] | GTTGGTGTAATGGGTCAGTATTAACTGTAAGCAACTCGTCGGTGG |
| stap17[49]-3[59] | AGATTAAGAGGAAGAGTAAGAAGAA |
| stap23[143]-6[142] | TTAAGTGTCCTGTCAGACTGATA |
| stap23[209]-5[213] | ATCTATTTGAAAACTTCCA |
| stap13[277]-1[286] | TATTTTGCGTATTGGGCTCGGAAAAACGCTCTACGTGG |
| stap22[110]-7[104] | AAGGATTAGGATTCAGGGTGGATGTTCGGAGTTAAAGGCCCTTGATAC |
| stap22[216]-4[213] | ATCATTCAATCAATTTAT |
| stap18[260]-5[248] | TCTTTAATGCGCGAACACAAAATTCAATTAATCG |
| stap14[307]-15[286] | CGGCCAACGCGCGGGGAGAGGCGGTTACACGCTTT |
| stap15[56]-1[62] | CTCCAACTTTCAACTAATGTG |
| stap3[60]-21[76] | GCCTTATGTTACTTAGCCGGAACGCTGACCAACTTTTACATGCCA |
| stap8[279]-11[279] | GTTGTAGGCCTGAGCAAACTAACCGCCA |
| stap4[307]-21[311] | TGGGGTCGGTGCTTGTTA |
| stap13[112]-17[118] | ACGTTAGCCCTCATAACGCCTAACCCATTTTCAGG |
| stap19[182]-23[188] | GAGGCGTCAAGCAATAGGAATGTACCGCTGTCCCG |
| stap14[104]-15[97] | GTAACGATCTAAATCGCGTCA |
| stap7[186]-8[175] | AGTTACCAGACCGTGTGATAAATAGCTATCT |
| stap0[283]-4[277] | GCGTTGAGAAGAGGCCGATTA |
| stap13[136]-13[135] | AGAGAGCATTGACAGGCACAGACAGTAAATGAATTTTCCCTC |
| stap5[91]-21[106] | GTGTACAATAAGGGGCTCAGTACCAGGCGG |
| stap18[41]-15[47] | AGCGGCAGCCCTGTAATACTTTAAAAATAGACCG |
| stap12[97]-8[98] | TTTCATTTAAAAACGAAAGAGCCCCCAGAAGGCTCTCTTAAA |
| stap17[77]-17[107] | TTTAGGAATACCACATTCAACTACACCACCC |
| stap23[157]-3[153] | TGCTCCTATTATTCTGAACCCCTGCAAACCATAGCAAGG |
| stap17[285]-3[300] | TGGCTGTAATAAAGACCAAGCACTAAATCG |
| stap2[121]-4[112] | ACGTATTAAAATTCAAAACACTCAATCGTCACTTTGAG |

Table S8: Table core staples sequences part 4.

| Position name | Sequence |
|---|---|
| stap1[224]-9[216] | GAAACAGATTAATTTTAGAACATTTTCAGGCTAAATTTAATGGGAATCA |
| stap22[69]-4[63] | TAGCGTCAACAGTTAAGCTAA |
| stap22[230]-7[230] | GCAAATCCCCTGGAGTGACTCCGCCTGATTGCTAGTAACGTC |
| stap2[205]-8[203] | GAAAGATTAAATCCTTGAAACACCGTTTGAA |
| stap8[202]-7[216] | ATACCGAAGTTTTAGTTAATTTCATCTT |
| stap13[70]-14[52] | TATGCGACGTTGGGAAGAAGTCAGGATTAGAG |
| stap18[275]-16[252] | TTGCGTAAGAAGATAGAACCCTTCTGCACTAACAACTA |
| stap15[70]-16[52] | TACGTTAGGTAGAAAGATTCGAAAGACTTCAA |
| stap15[133]-2[122] | GAGGCAGATAAATCCTCATCCCAATAGGGTAGCATAAAT |
| stap21[77]-14[84] | GAGGGGGTAATATTAACCGAAAGGCGCATTGTGTCGAACTAATATACCA |
| stap2[300]-15[307] | GACGGGGGGAAACCTGTCGTG |
| stap17[108]-16[84] | TCATGTACCGTAACACTGAGTTTGAACAACAT |
| stap5[105]-13[111] | TTGCGGGTCTTTGAGCAAAAGACAACTTTTTCCAG |
| stap8[265]-8[245] | TCTTTGATTAAAACAGAAATA |
| stap19[203]-19[202] | AGAACGCCTAATTTACGAGGCCCAATAGTTTTCAGCTAATGC |
| stap1[21]-12[35] | TAATGCCCTGTAGCACAACCCGTGATAAGAGGTCAATGTTTT |
| stap15[14]-14[21] | GGATAGGTCACGTTGATTCTC |
| stap5[214]-12[203] | ATCCCTTAGAGACGCTGATACAAATTCTTGC |
| stap17[119]-4[119] | GATAGCAACCCTCATTAAACGACAGAGG |
| stap8[139]-10[119] | CCCCTTATTTTATCTCGGTAGACGAATAATAATTT |
| stap15[287]-3[282] | CCAGTCGAAAGCCGGCGAATGAGC |
| stap23[189]-0[188] | CCAAAATAACCGAGCTGGCATAGCCAGCG |
| stap12[307]-13[307] | CAGCTGATTGCCCTGCCAGGGTGGTTTT |
| stap18[82]-17[76] | CAAAAGGAATTACGAGGCATCCCATCAGTTGAGA |
| stap0[251]-5[265] | TTGCTTCTTATTTGCACGTAGTAATAACCCGCGCTAAGAACG |
| stap8[117]-22[111] | TTCGAGTGCGCCGCTTGCAGTTCTAAGCTCAAGAG |
| stap10[76]-0[49] | AACGAGTAGTAACAGTTGATTCCCAATTATACATTGGGTGAG |
| stap16[83]-8[77] | TATTACAATAAAACGAAATCCGTATCATGCGAAACATTCAGTCCAAATC |
| stap22[82]-5[90] | TGTTTACCAAGCTTTCTCAGTAGGCTGATGAACG |
| stap3[133]-17[133] | GCACCATGGCTTTTGATGACCAGTAAAGC |
| stap19[224]-19[247] | AATAGATAAGTCGTCAGTTATAGC |
| stap10[118]-8[118] | TTTCACGAGCCTTTAATTGTAAGCTTGCT |
| stap23[251]-0[252] | GTGCGGCCCGGGAGAAGAGGCGTATAACC |
| stap7[231]-11[237] | AGATGAATGCGTAGCTACCATTTCTGAAGATGATG |
| stap20[153]-16[147] | GAGTAACTAAGTTTTAAGCGTGCAGTCTATATTCA |
| stap21[44]-5[48] | ATCCCAAACATTATGACAACCGAATTAGCAAAA |
| stap14[148]-10[140] | GCCGCCGCCCCGCCACACCCTCACAGAGCCGAGCCAC |
| stap22[258]-15[265] | TGCAACAGAACCTCATTCATTTAGCTTTAACGTATCGCAGTAATAAAAG |

# 3 Supplementary figures

## 3.1 AFM images of the 24HB without extensions

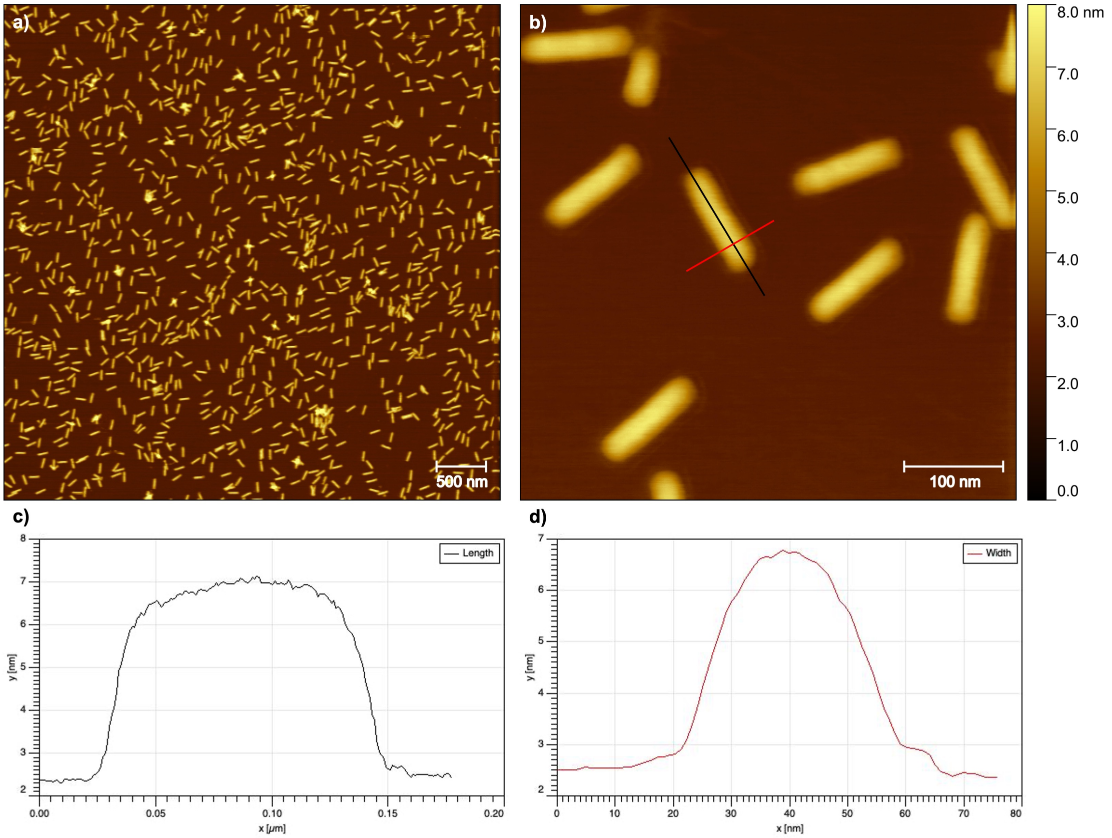


Figure S5: Representative AFM images of the 24HB in its non-extended form designed to incorporate linker strands and fluorophore binding sites. DNA origami folded in 1× TAE buffer with 20 mM $MgCl_2$. a) large-area scan (5 × 5 $\mu$m) showing homogeneous distribution of the folded structures, b) zoom-in scan (500 × 500 nm) highlighting cross-sectional profiles of an individual folded structure, c) length profile along a representative folded structure [blue line in part b)], and (d) lateral cross-section analysis along the red line in part b) illustrating the expected height and width of the folded structure.

## 3.2 AFM images of the 24HB extended with Cy3 and 6-FAM dyes

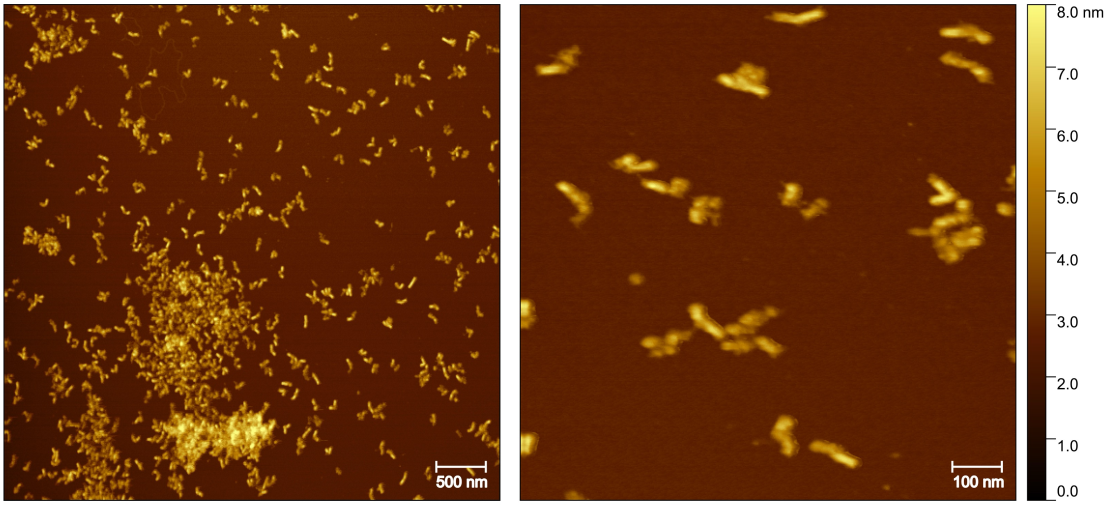


Figure S6: Representative AFM images of the annealed 24HB structures with attached Cy3, 6-FAM and linker strands using 1× TAE with 10 mM $MgCl_2$ FOB. a) large-area scan (5 × 5 $\mu$m) showing aggregations and b) zoom-in scan (500 × 500 nm) showing misfolded structures.

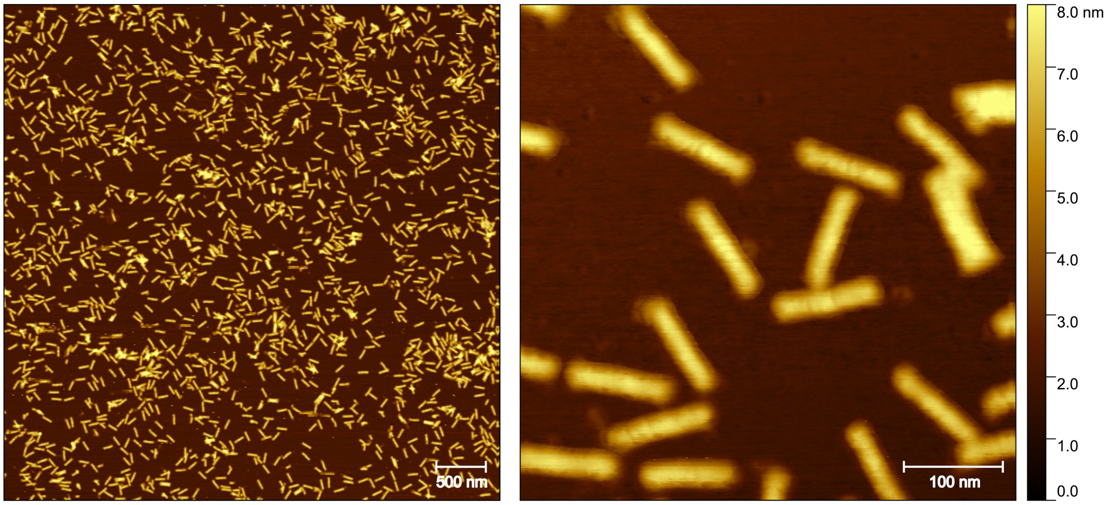


Figure S7: Representative AFM images of the annealed 24HB structures with attached Cy3, 6-FAM and linker strands using 1× TAE with 20 mM $MgCl_2$ FOB. The structures are well-folded and show homogeneous distribution across the surface, indicating improved structural stability compared to samples prepared at a lower $MgCl_2$ concentration. a) large-area scan (5 × 5 $\mu$m) and b) zoom-in scan (500 × 500 nm).

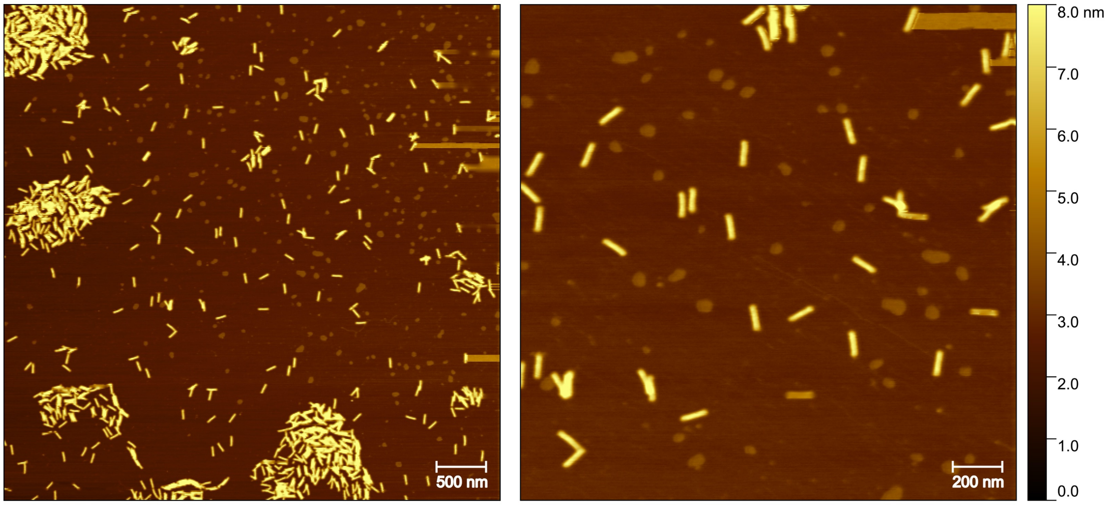


Figure S8: Representative AFM images of the annealed 24HB structures with attached Cy3, 6-FAM and linker strands using 1× TAE with 40 mM $MgCl_2$ FOB. Individual structures appear properly folded, with a tendency to associate into localized agglomerates, forming island-like domains on the mica surface. a) large-area scan (5 × 5 $\mu$m) and b) zoom-in scan (1 × 1 $\mu$m).

### 3.3 Fluorescence microscopy (FM) images

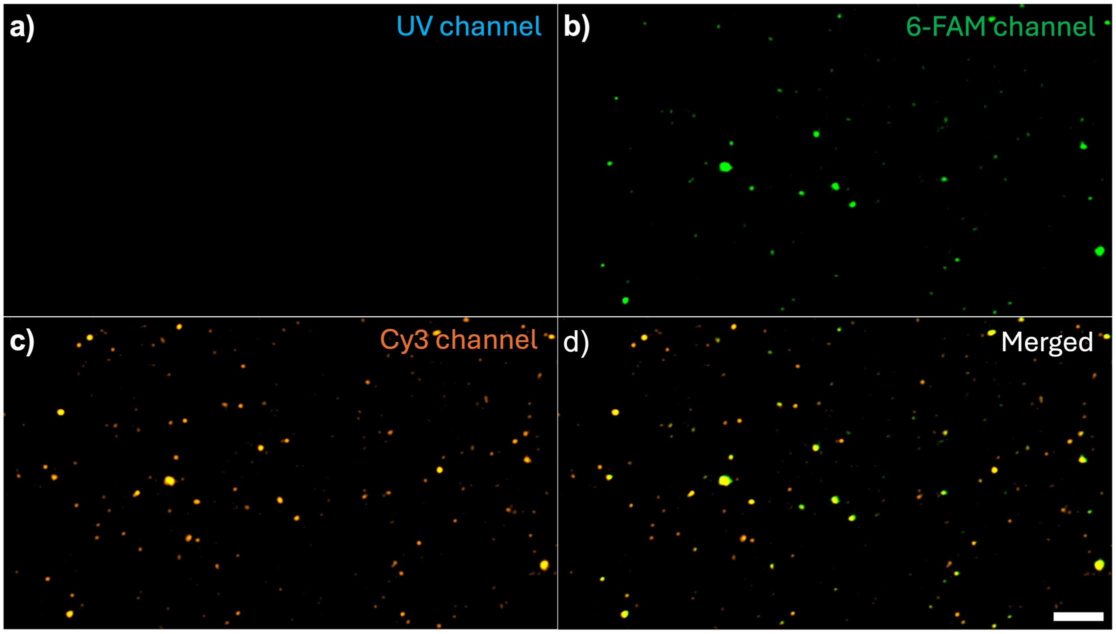


Figure S9: Representative FM images of the 24HB structures folded in 1× TAE buffer with 20 mM $MgCl_2$. a) UV channel (excitation at 365 nm), b) 6-FAM channel (excitation at 460 nm), c) Cy3 channel (excitation at 550 nm) and d) merged image. Scale bar: 4$\mu$m.

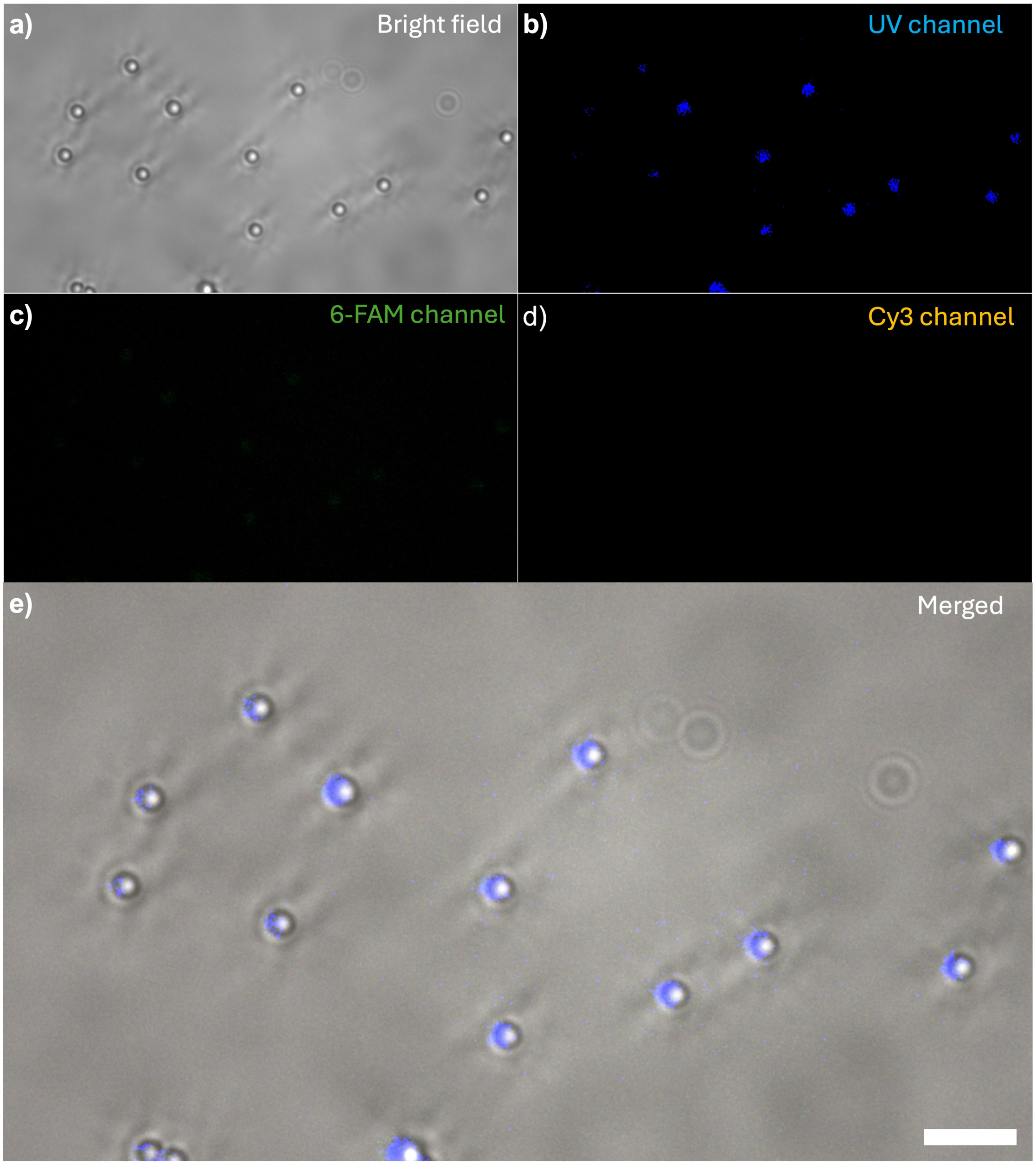


Figure S10: Representative FM images of plain streptavidin-coated polystyrene microspheres (Sa-Ps). a) bright field, b) UV channel (excitation at 365 nm) showing autofluorescence emission of Sa-PS, c) 6-FAM channel (excitation at 460 nm), d) Cy3 channel (excitation at 550 nm) and e) merged image. Scale bar: 4$\mu$m.

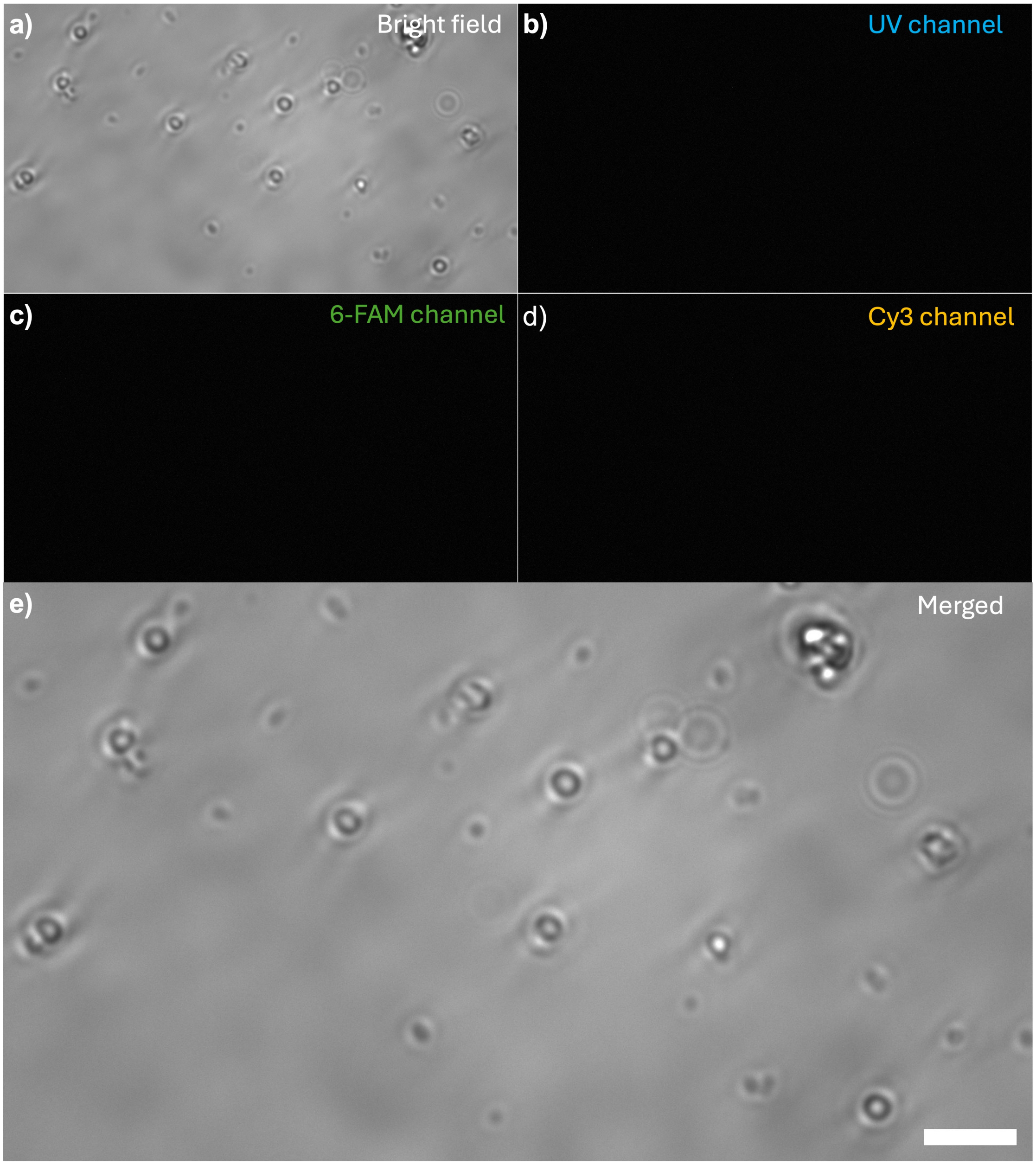


Figure S11: Representative FM images of plain azide-coated magnetic microspheres. a) bright field, b) UV channel (excitation at 365 nm), c) 6-FAM channel (excitation at 460 nm), d) Cy3 channel (excitation at 550 nm) and e) merged image. Scale bar: 4$\mu$m.

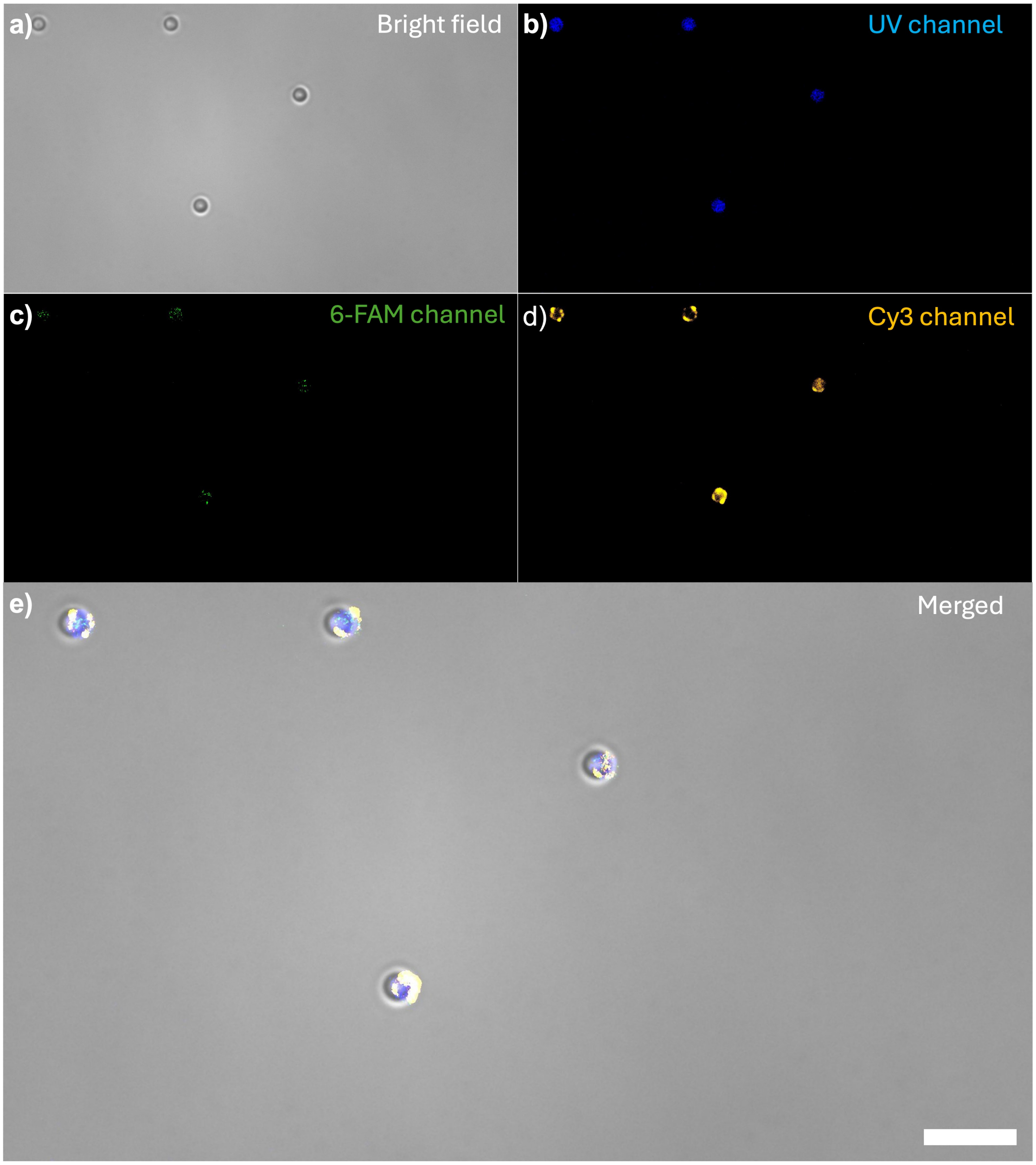


Figure S12: Representative FM images of streptavidin-coated polystyrene microspheres incubated with the 24HB at the ratio of 1:100. a) bright field, b) UV channel (excitation at 365 nm), c) 6-FAM channel (excitation at 460 nm), d) Cy3 channel (excitation at 550 nm) and e) merged image. Scale bar: 4$\mu$m.

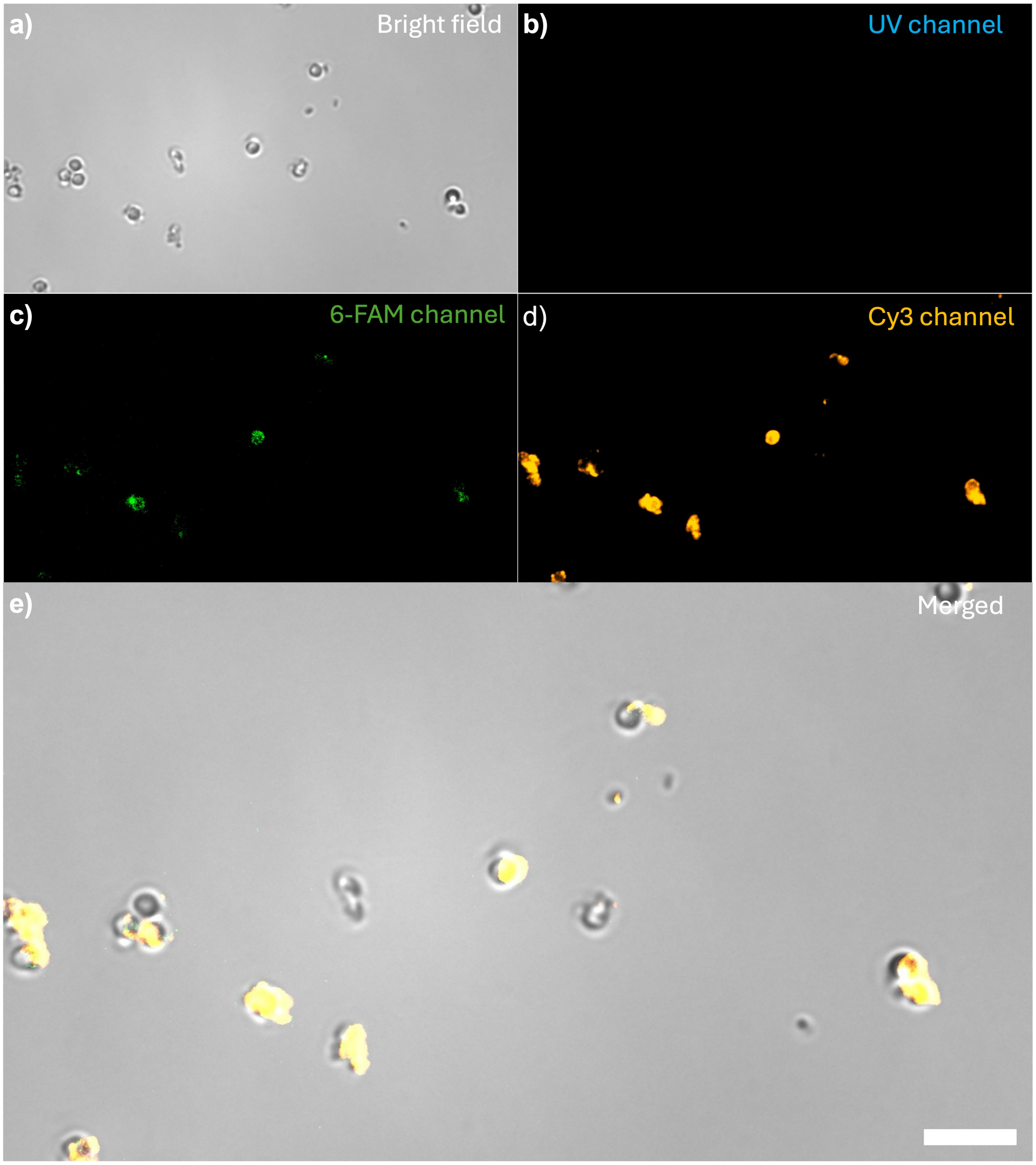


Figure S13: Representative FM images of azide-coated magnetic microspheres incubated with the 24HB at the ratio of 1:100. a) bright field, b) UV channel (excitation at 365 nm), c) 6-FAM channel (excitation at 460 nm), d) Cy3 channel (excitation at 550 nm) and e) merged image. Scale bar: $4 \mu m$.

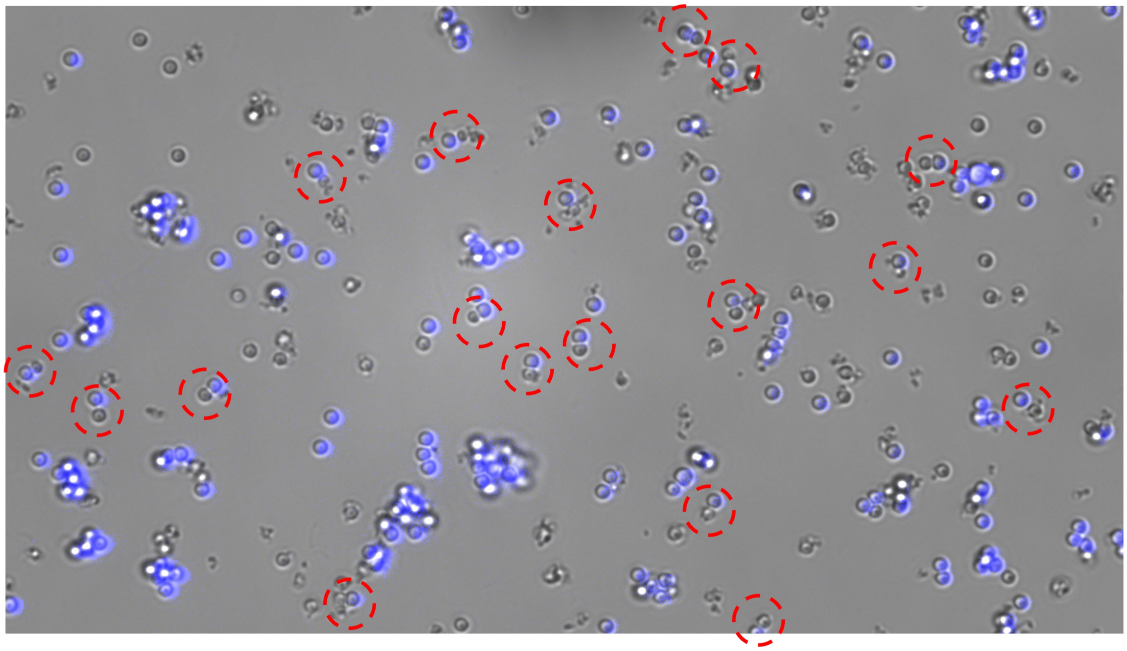

Figure S14: Representative distribution of the heterodimer formation at the 1:1:100 microsphere-to-24HB ratio. Quantification of hybrid microsphere assemblies was carried out using correlations between the fluorescence (excitation at 365 nm) and bright-field images across 10 images ($\sim$ 88.9 $\mu$m $\times$ 50.0 $\mu$m each). Red dashed circles indicate identified heterodimers.

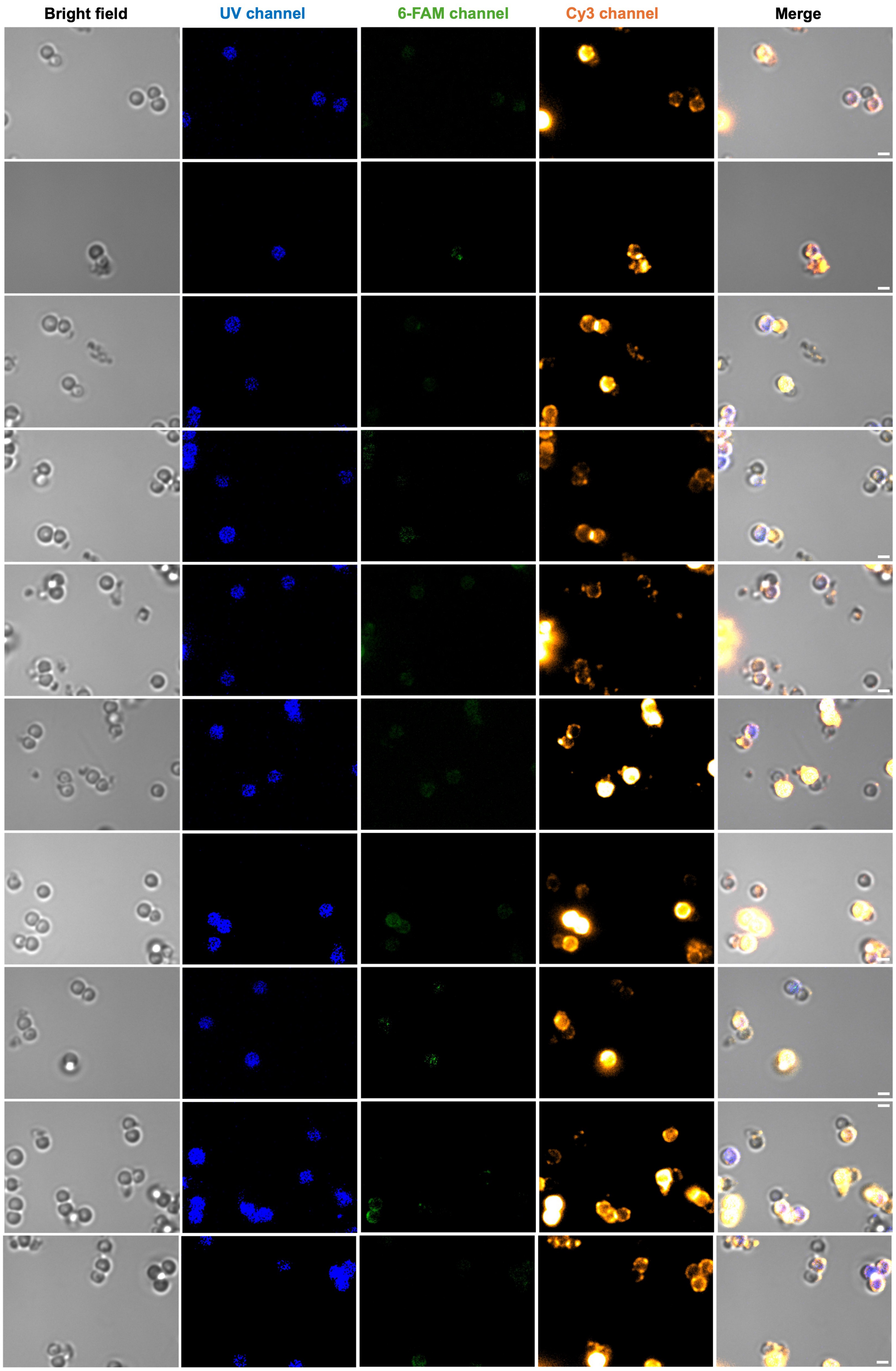


Figure S15: Representative FM images of the selected 1:1:100 samples. Scale bar: 1$\mu$m

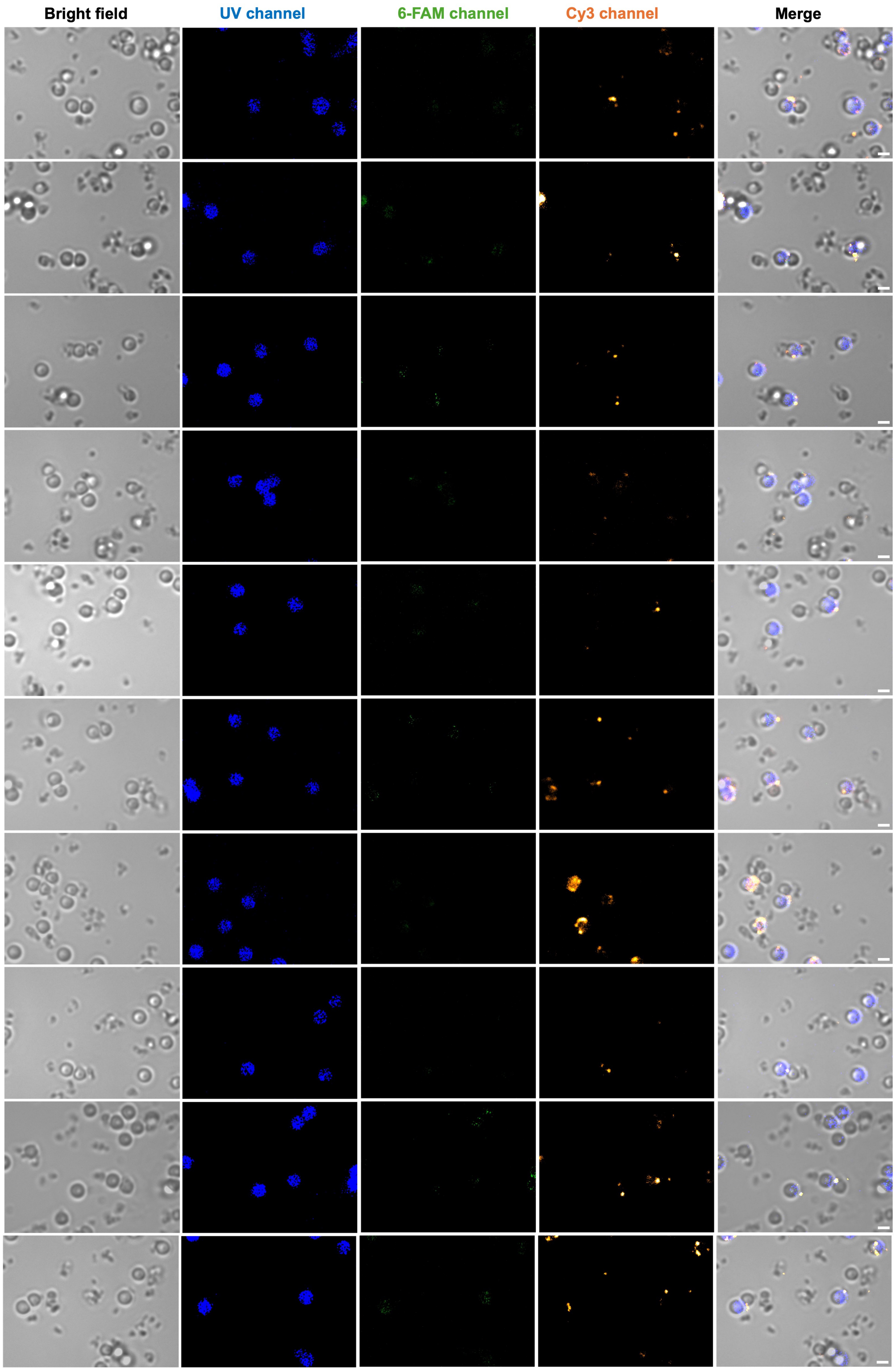


Figure S16: Representative FM images of the selected 1:1:10 samples. Scale bar: 1$\mu$m

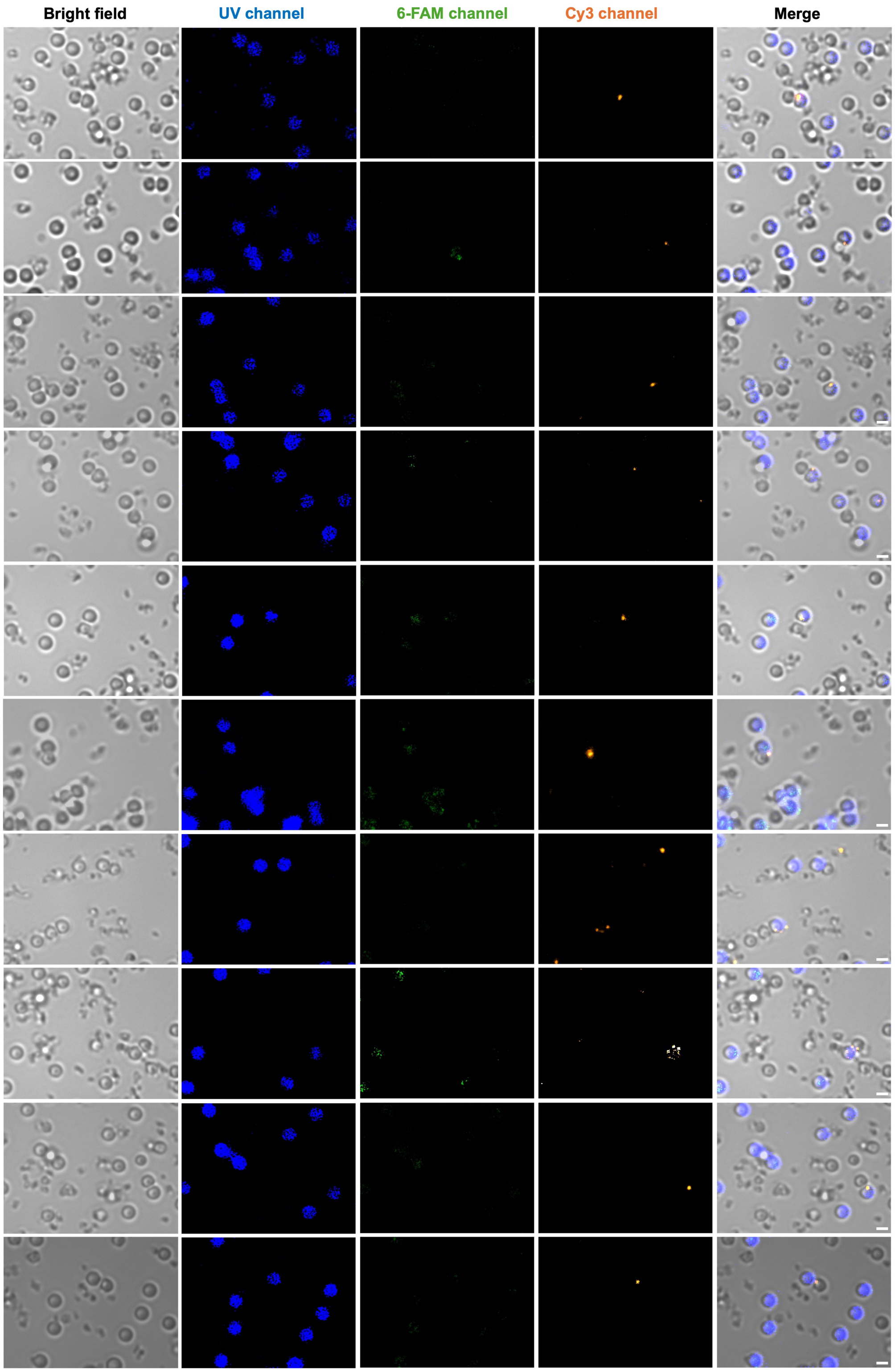


Figure S17: Representative FM images of the selected 1:1:1 samples. Scale bar: $1 \mu m$

## 3.4 TEM images

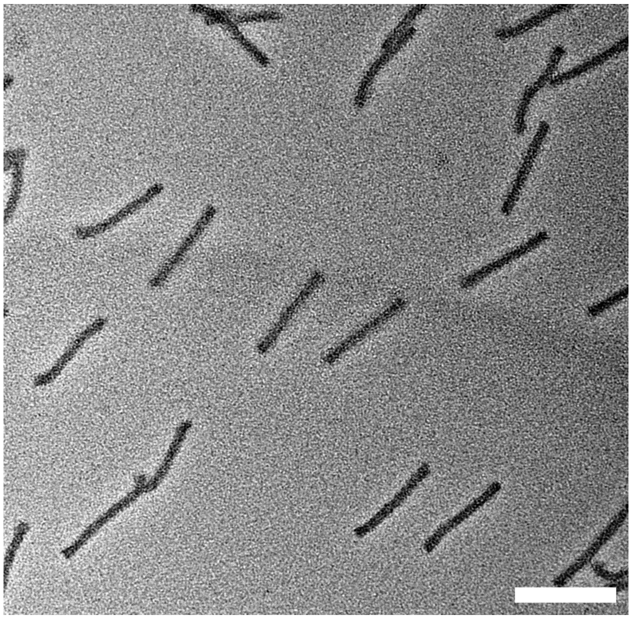

Figure S18: Representative TEM image of the 24HB. Scale bar: 100 nm.

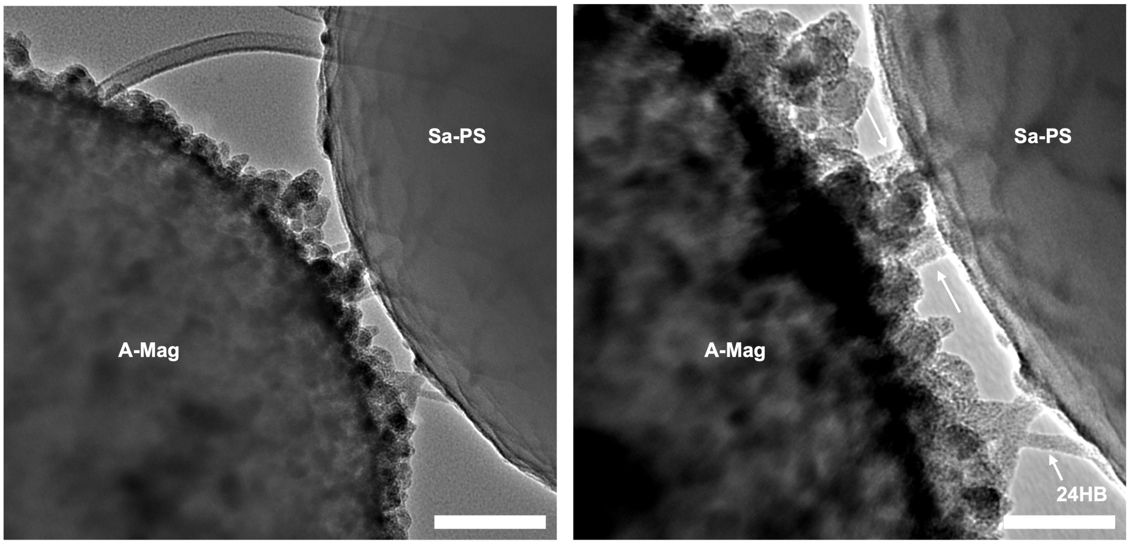


Figure S19: Representative TEM images of the heterodimer formation, showing the 24HB bridging the microspheres (see the white arrows in the right-side image). Sa-PS: streptavidin-coated polystyrene microspheres, A-Mag: azide-coated magnetic microspheres. Scale bars: 100 nm (left) and 50 nm (right).

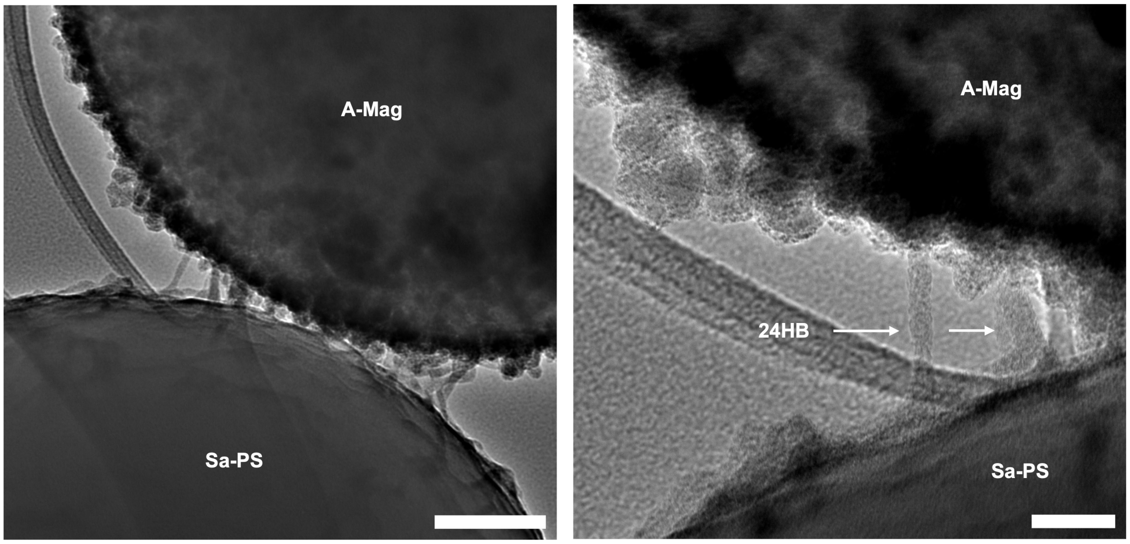


Figure S20: Representative TEM images of the heterodimer formation, showing the 24HB bridging the microspheres (see the white arrows in the right-side image). Sa-PS: streptavidin-coated polystyrene microspheres, A-Mag: azide-coated magnetic microspheres. Scale bars: 100 nm (left) and 50 nm (right).

## 3.5 Bistability of heterodimers optically trapped in 2D.

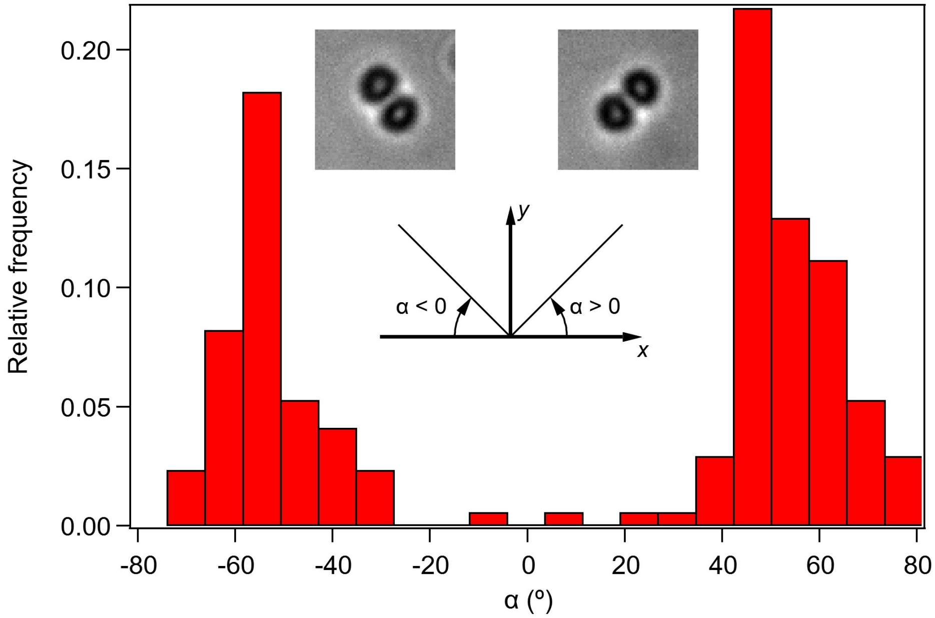


Figure S21: The normalized histogram of in-plane orientation angles $\alpha$ of an optically trapped heterodimer observed during its 2D surface-assisted optical manipulation in the vicinity of the sample chamber wall (see Figure 4b of the main article and supporting video "2D Dimer Trapping" for additional details). The strongly bimodal nature of the histogram, which peaks at angles $\alpha_{+}$=45°, $\alpha_{-}$=54°, directly reflects the bistable nature of the 2D optical confinement of the heterodimer in the linearly polarized trapping beam, with the principal orientations of the heterodimer aligned along the direction of the trapping beam polarization ($\alpha_{-}$) or perpendicular to it ($\alpha_{+}$). The width of the two histogram peaks is then determined by the combined effect of thermal position fluctuations driven by collisions of the heterodimer with the ambient liquid molecules and of hydrodynamic forces induced by translating the sample chamber past the stationary confined heterodimer (see also the discussion in the main article).

## 3.6 Effects of prolonged exposure to the trapping light on the fluorescence emission of optically trapped heterodimers

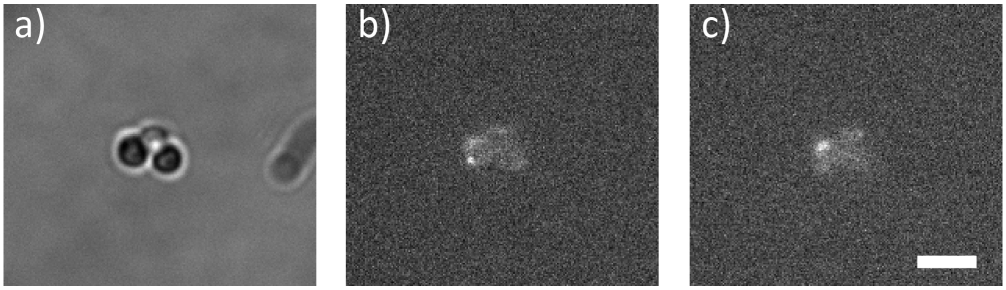


Figure S22: Influence of prolonged exposure to the infrared trapping light on the stability of fluorescence emission from a heterodimer optically confined in 2D. a) Bright-field image of the heterodimer confined near the top wall of the sample chamber. b) Fluorescence emission recorded from the same heterodimer within 2 seconds after the initial confinement. c) Fluorescence emission recorded from the same heterodimer after additional 20 seconds of continuous exposure to the infrared trapping beam with the power at the sample plane set to $\sim$ 31 mW. Image exposure time: 12.5 ms in part a), 500 ms in parts b) and c). Scale bar: 2 $\mu$m.

# References

(1) Kuzyk, A.; Schreiber, R.; Fan, Z.; Pardatscher, G.; Roller, E.-M.; Högele, A.; Simmel, F. C.; Govorov, A. O.; Liedl, T. DNA-based Self-assembly of Chiral Plasmonic Nanostructures with Tailored Optical Response. *Nature* **2012**, *483*, 311–314.

(2) Kim, D.-N.; Kilchherr, F.; Dietz, H.; Bathe, M. Quantitative Prediction of 3D Solution Shape and Flexibility of Nucleic Acid Nanostructures. *Nucleic Acids Research* **2011**, *40*, 2862–2868.
